# Dependence of thick filament structure in relaxed mammalian skeletal muscle on temperature and interfilament spacing

**DOI:** 10.1085/jgp.202012713

**Published:** 2021-01-08

**Authors:** Marco Caremani, Luca Fusi, Marco Linari, Massimo Reconditi, Gabriella Piazzesi, Thomas C. Irving, Theyencheri Narayanan, Malcolm Irving, Vincenzo Lombardi, Elisabetta Brunello

**Affiliations:** 1 PhysioLab, University of Florence, Florence, Italy; 2 Randall Centre for Cell and Molecular Biophysics, King’s College London, London, UK; 3 Consorzio Nazionale Interuniversitario per le Scienze Fisiche della Materia, Firenze, Italy; 4 Center for Synchrotron Radiation Research and Instrumentation and Department of Biological Sciences, Illinois Institute of Technology, Chicago, IL; 5 European Synchrotron Radiation Facility, Grenoble, France

## Abstract

Contraction of skeletal muscle is regulated by structural changes in both actin-containing thin filaments and myosin-containing thick filaments, but myosin-based regulation is unlikely to be preserved after thick filament isolation, and its structural basis remains poorly characterized. Here, we describe the periodic features of the thick filament structure in situ by high-resolution small-angle x-ray diffraction and interference. We used both relaxed demembranated fibers and resting intact muscle preparations to assess whether thick filament regulation is preserved in demembranated fibers, which have been widely used for previous studies. We show that the thick filaments in both preparations exhibit two closely spaced axial periodicities, 43.1 nm and 45.5 nm, at near-physiological temperature. The shorter periodicity matches that of the myosin helix, and x-ray interference between the two arrays of myosin in the bipolar filament shows that all zones of the filament follow this periodicity. The 45.5-nm repeat has no helical component and originates from myosin layers closer to the filament midpoint associated with the titin super-repeat in that region. Cooling relaxed or resting muscle, which partially mimics the effects of calcium activation on thick filament structure, disrupts the helical order of the myosin motors, and they move out from the filament backbone. Compression of the filament lattice of demembranated fibers by 5% Dextran, which restores interfilament spacing to that in intact muscle, stabilizes the higher-temperature structure. The axial periodicity of the filament backbone increases on cooling, but in lattice-compressed fibers the periodicity of the myosin heads does not follow the extension of the backbone. Thick filament structure in lattice-compressed demembranated fibers at near-physiological temperature is similar to that in intact resting muscle, suggesting that the native structure of the thick filament is largely preserved after demembranation in these conditions, although not in the conditions used for most previous studies with this preparation.

## Teaser

Caremani et al. use x-ray diffraction and interference to characterize the structure of thick filaments in relaxed demembranated and intact resting mammalian muscle, revealing two closely spaced axial periodicities as well as structural changes that occur when filament activation is mimicked by cooling below near-physiological temperature.

## Introduction

Muscle contraction is driven by relative sliding between thick (myosin-containing) and thin (actin-containing) filaments. Myosin head or motor domains on the surface of the thick filaments bind to actin in the thin filaments, change shape to drive filament sliding, and then detach in a mechano-chemical cycle coupled to ATP hydrolysis. In resting muscle, contraction is inhibited by tropomyosin, a thin filament component that blocks the myosin-binding sites on actin. When muscle cells are electrically stimulated, calcium ions are released from intracellular stores and bind to another thin filament protein, troponin, causing a structural change in the thin filaments that displaces tropomyosin from its blocking position (Huxley, 1973; Parry and Squire, 1973).

More recently it became clear that the thick filament also plays a significant role in the regulation of contraction. The myosin heads in resting muscle are not available for actin binding because they are locked in a helical array on the surface of the thick filaments (Huxley, 1973; Haselgrove, 1975; Irving, 2017). EM of isolated thick filaments showed that the heads can fold back onto their tails in a conformation called the interacting heads motif (IHM), stabilized by head–tail interactions, head–head interactions, and probably interactions between myosin and other thick filament components including titin and myosin-binding protein-C (MyBP-C; Woodhead et al., 2005). The net effect is to inhibit ATP hydrolysis and actin binding by the myosin heads, and this state of myosin has also been called the super-relaxed or SRX state (Woodhead et al., 2005; Stewart et al., 2010; Irving, 2017).

Much less is known about the structural basis of thick filament–based regulatory mechanisms than of their thin filament counterparts. The structure of the thick filament varies along its length; this is related to the packing of the myosin tails into a bipolar structure tapering at the tips, the presence of MyBP-C in the central region of the half-filament—the so-called C-zone (Bennett et al., 1986)—and the different super-repeat of titin, the modular thick filament scaffold protein in different regions of the half-filament (Labeit and Kolmerer, 1995; Tonino et al., 2019; Bennett et al., 2020). Neither the packing of the myosin tails nor the interactions between myosin, titin, and MyBP-C have been described at molecular resolution in vertebrate thick filaments. Finally, the thick filament exhibits two closely spaced axial periodicities in resting muscle, and the shorter of these periodicities increases when muscle is activated (Huxley and Brown, 1967; Haselgrove, 1975; Linari et al., 2015). The molecular bases of the two periodicities and the changes on activation are also unknown.

The complexity of the thick filament structure and the lack of detailed information about the interactions between its component proteins have probably impeded understanding of the signaling pathways underlying thick filament regulation. A more fundamental obstacle, however, is that thick filament regulation cannot be fully reconstituted in isolated filaments or their protein components because its signaling pathways depend on the activation of neighboring thin filaments and on active force generation per se (Reconditi et al., 2011; Linari et al., 2015; Irving, 2017; Piazzesi et al., 2018). Physiological signaling between the regulatory states of the thin and thick filaments depends on the integrity of the native lattice of thick and thin filaments in the muscle cell.

For these reasons, we chose to investigate thick filament regulation in demembranated muscle fibers, in which the native filament lattice is preserved, but the interfilament spacing and the solution bathing the filaments can be controlled. We used synchrotron-based x-ray diffraction and interference to characterize the changes in thick filament structure induced by cooling mammalian muscle fibers from the near-physiological temperature of 35 to 10°C in relaxing conditions. This protocol disrupts the inhibited IHM or SRX state of the thick filament by altering the bound nucleotide in the active site of the myosin head domains to change the conformation of the myosin heads. The ADP.Pi or “switch-2 closed” state is favored at 35°C, and this is required for the folded and helically ordered IHM state, whereas the ATP or “switch-2 open” state is favored at 10°C (Wray, 1987; Malinchik et al., 1997; Xu et al., 1997, 1999, 2003; Caremani et al., 2019).

Although some of the effects of cooling on the x-ray diffraction patterns from relaxed demembranated muscle fibers have been reported previously (Wray, 1987; Malinchik et al., 1997; Xu et al., 1997, 1999, 2003), the improved spatial resolution of current synchrotron x-ray beamlines and detectors makes it possible to resolve the interference fine structure of the axial x-ray reflections with unprecedented precision, allowing the location of the diffracting structures within the half-thick filament to be determined, as shown below. Moreover, previous studies did not control for the expansion of the filament lattice on demembranation, which can increase the distance between the surfaces of the thick and thin filaments by almost a factor of two at low temperature, as also shown below. Such a large increase in interfilament separation is likely to disrupt signaling between the thin and thick filaments, as mediated, for example, by binding of the N terminus of MyBP-C to the thin filaments (Moos et al., 1975; Luther et al., 2011). We therefore controlled interfilament spacing by osmotic compression of the filament lattice by Dextran T-500, a long-chain polymer that does not penetrate the lattice, and made all the x-ray measurements both in the absence and presence of Dextran at a concentration at which the interfilament spacing is slightly less than that in intact resting muscle. Finally, as a more general test of the preservation of thick filament structure and regulation in demembranated muscle fibers, we compared each x-ray parameter with that measured in intact resting extensor digitorum longus (EDL) muscle of the mouse across a similar temperature range (Caremani et al., 2019). The results reveal some novel features of thick filament structure and how they change on thick filament activation and show that these structural and regulatory properties are largely preserved in demembranated fibers from mammalian muscle in the presence of Dextran, but only at higher temperatures that were not generally used in previous studies.

## Materials and methods

### Experiments on demembranated muscle fiber bundles

Glycerinated skinned fiber segments were prepared from psoas muscles of adult male New Zealand white rabbits as described previously (Linari et al., 2007). In short, rabbits were killed by injection of an overdose of sodium pentobarbitone (150 mg kg^−1^) in the marginal ear vein, in accordance with protocols approved by the Italian Ministry of Health (authorization no. 956/2015 PR) in compliance with the Italian regulation on animal experimentation, Decreto Legislativo 26/2014, and the EU regulation (directive 2010/63). Bundles of 70–150 fibers were stored in skinning solution (Linari et al., 2007) containing 50% glycerol at −20°C for 1–2 wk, and smaller bundles of fibers 5–6 mm long were dissected on the day of the experiment. The ends of the bundles were held in T-shaped aluminum clips for attachment between coaxial hooks, one of which was attached to a strain gauge force transducer (AE801; Sensor) on an apparatus providing rapid solution exchange and temperature control (Linari et al., 2007) modified for synchrotron x-ray experiments. For the x-ray experiments, bundles of 10–15 fibers with a maximum width of 425 ± 15 µm and 2–3 fibers deep were mounted in the experimental trough, and the sarcomere length was set to 2.49 ± 0.05 µm (mean ± SD, *n* = 10 bundles). For the Ca^2+^-titration experiments in Fig. S10, single fibers (length, 3.6 ± 0.6 mm; width, 89 ± 11 µm; cross-sectional area, 6,300 ± 1,300 µm^2^) or bundles of 4–5 fibers were used, and the sarcomere length was set to 2.49 ± 0.04 µm (mean ± SD, *n* = 7). Addition of 5% Dextran to the relaxing solution reduced the fiber width by 13.7% ± 3.0% and the cross-sectional area by 25.4% ± 5.3% (mean ± SD, *n* = 5 fibers). Before each experiment, the ends of the bundle were fixed with glutaraldehyde and glued to the aluminum clips with shellac dissolved in ethanol. Force and x-ray acquisition timing were collected and analyzed using custom software routines written in LabVIEW (National Instruments).

### Solutions

The solution compositions were calculated using software kindly provided by Prof. Earl Homsher (University of California, Los Angeles, Los Angeles, CA). In the x-ray experiments, relaxing solution contained 100 mM N-tris(hydroxymethyl)methyl-2-aminoethanesulfonic acid, 5.55 mM Na_2_ATP, 19.11 mM Na_2_Creatine phosphate, 10 mM reduced glutathione, 7.75/7.65/7.45 mM MgCl_2_ (12°C/25°C/35°C), and 26/22.25/19 mM EGTA (12°C/25°C/35°C). All solutions contained 5 mM MgATP, 1.1 mM free Mg^2+^, and 190 mM ionic strength, with pH set to 7.1 at 12°C/25°C/35°C. Solutions for the experiments at 20°C were prepared by mixing the solutions for 12°C and 25°C and those at 30°C by mixing the solutions for 25°C and 35°C. Where specified, the osmotic agent Dextran T500 (5% wt/vol; Pharmacia Biotech) was added to the solutions. The Dextran concentration used here (5%) was determined in pilot x-ray experiments as that required to compress the filament lattice spacing to a value close to that measured in resting EDL muscle of the mouse. Composition of the solutions used for the force/pCa titrations at 12°C were as published (Linari et al., 2007). Relaxing and activating solutions were mixed to obtain a series of partial activating solutions with the required free calcium ion concentrations. Maximum active force was not affected by the addition of 5% Dextran.

### Ca^2+^ titration of isometric force

Single fibers or small bundles prepared as described above were activated in a multidrop apparatus using a temperature jump technique (Linari et al., 2007); they were kept in preactivating solution at low temperature (1°C) for 1 min, then transferred to activating solution at 1°C, in which little force was developed. When this force became steady (within 3 s), the fiber was transferred to activating solution at the test temperature, 12°C. After activation, the fiber was transferred to relaxing solution. This procedure has been shown to prevent the development of sarcomere nonuniformities related to diffusion-limited activation across the fiber (Linari et al., 2007). The maximal isometric force developed at saturating calcium concentration (pCa 4.7) was 184 ± 44 kPa (mean ± SD, *n* = 5 fibers).

### Experiments on intact EDL muscles

The x-ray diffraction data obtained in relaxed demembranated fibers from rabbit psoas muscle were compared with analogous data from resting mouse EDL muscles in the same temperature range. Some of the EDL data were presented previously in the context of a comparison with those obtained during active isometric contraction at the same temperatures (Caremani et al., 2019). Here, we present additional analysis of the x-ray diffraction data from resting mouse EDL muscle obtained in those experiments. Male mice (*Mus musculus*, strain C57BL/6) aged 4–6 wk were sacrificed by cervical dislocation after inhalation of isoflurane according to both the Italian regulation on animal experimentation (authorization no. 956/2015 PR), in compliance with Decreto Legislativo 26/2014 and the EU regulation (directive 2010/63), and the protocols approved by the Illinois Institute of Technology Institutional Animal Care and Use Committees. The EDL muscle was dissected from the hind limbs and mounted in a trough containing physiological solution between a fixed hook and the lever of a motor/force transducer system (300C-LR; Aurora Scientific) mounted on a micromanipulator for adjustment of the muscle length. The physiological solution (composition in mM: 119 NaCl, 4.7 KCl, 2.5 CaCl_2_, 1.0 MgSO_4_, 25 NaHCO_3_, 1.2 KH_2_PO_4_, and 1.1 glucose, pH 7.4, at 24–26°C) was equilibrated with carbogen (95% O_2_ and 5% CO_2_), and the temperature of the bathing solution was selected and kept constant (±0.2°C) by means of a servo-controlled thermoelectric module. Initial muscle length was adjusted to that at which isometric tetanic force was the maximum (*L*_0_), corresponding to a sarcomere length of ∼2.6 µm (Caremani et al., 2019). Six muscles were used, with length *L*_0_ = 9.5 ± 1.6 mm and wet weight 10.5 ± 1.2 mg (mean ± SD).

### X-ray data collection

Bundles of demembranated fibers from rabbit psoas muscle were mounted horizontally at beamline ID02 (Narayanan, 2014) of the European Synchrotron Radiation Facility (ESRF), which provided up to 2 × 10^13^ photons s^−1^ at 0.1-nm wavelength, with beam size measured on the detector approximately 144 × 128 µm (horizontal × vertical, full width at half-maximum, 3-m camera length) or 155 × 130 µm at the detector (6-m camera length). The measured beam size is convoluted by the ∼85-µm point spread function of the detector. The beam was attenuated for bundle alignment. To reduce scattering from the solution, x-ray exposures were acquired while the bundle was in air in a small chamber at the test temperature. To minimize radiation damage, x-ray exposure was limited to the data collection period (10 or 20 ms) using a fast electromagnetic shutter (NM Laser Products, Inc.), and the bundle was moved horizontally along its axis by 200–400 µm between exposures. Total exposure time varied from 200 to 1,000 ms in each bundle before radiation damage occurred. The x-ray diffraction patterns were recorded using the FReLoN detector, a fiber-optically–coupled charge-coupled device (CCD) detector (Sztucki et al., 2010; Narayanan, 2014), active area 100 × 100 mm^2^, 2,048 × 2,048 pixels. Before readout of the CCD, pixels were binned by eight in the direction perpendicular to the fiber axis to increase the signal-to-noise ratio (S/N) while preserving the fine structure on the meridional reflections. Camera length was set to 6 m or 3 m to record the interference fine structure of the meridional reflections up to the third- or the sixth-order myosin-based reflection, respectively. The x-ray data are presented from 10 bundles, 5 at the longer camera length and 5 at the shorter camera length.

EDL muscles were vertically mounted at the BioCAT beamline 18ID (Fischetti et al., 2004) of the Advanced Photon Source (APS), which provided ∼10^13^ photon s^−1^ at 0.1-nm wavelength in a beam size of ∼350 × 350 µm (horizontal × vertical, full width at half-maximum, 3.5-m camera length) at the sample and ∼160 × 65 µm at the detector. The beam was attenuated for muscle alignment, and the trough was oscillated in the beam during the exposure to spread the x-rays over the sample and reduce radiation damage. The x-ray patterns were collected on a high-sensitivity, high–spatial resolution, two-chip CCD detector (PCCD1680; Aviex), active area 80 × 160 mm^2^, 2,084 × 4,168 pixels, and point spread function 65 µm.

### Data analysis

The x-ray diffraction data were analyzed using the SAXS package (P. Boesecke, ESRF, Grenoble, France), Fit2D (A. Hammersley, ESRF), and IgorPro (WaveMetrix, Inc.). Two-dimensional patterns were centered and aligned using the equatorial 1,0 or 1,1 reflections, then mirrored horizontally and vertically. The equatorial intensity distribution was determined by integrating from 0.0036 nm^−1^ on either side of the equatorial axis, and the intensities of the 1,0 and 1,1 reflections were determined by fitting four Gaussian peaks (1,0; Z line; 1,1 and 2,0; see Fig. 2, A and B). The distribution of diffracted intensity along the meridional axis of the x-ray pattern (parallel to the fiber axis) was calculated by integrating from 0.012 nm^−1^ on either side of the meridian for the myosin-based M1 to M6 reflections and troponin T1 reflection. Background intensity distributions were fitted by a convex hull, a line below all the points in the intensity distribution that contains no concavities; the small background remaining when the convex hull had been subtracted was removed using the intensity from a nearby region of the x-ray pattern containing no reflections or with a linear fit. Integrated intensities were obtained from the following axial regions: M1, 0.021–0.025 nm^−1^; T1, 0.025–0.028 nm^−1^; M2L, 0.040–0.045 nm^−1^; M2H, 0.046–0.049 nm^−1^; M3, 0.066–0.072 nm^−1^; M4L, 0.0860.092 nm^−1^; M4H, 0.092–0.095 nm^−1^; M5L, 0.108–0.112 nm^−1^; M5H, 0.114–0.118 nm^−1^; and M6, 0.137–0.142 nm^−1^, where L and H denote low- and high-angle components of each M reflection (see Results).

The cross-meridional width of the M2, M3, and M6 meridional reflections (see Fig. S2) was measured by integrating the 2-D patterns within the above integration limits parallel to the equatorial axis. The resulting intensity distributions were fitted with two Gaussian functions with the same center and widths that differed by about one order of magnitude; the wider function was considered to be background, and the narrower one was used to determine the cross-meridional width of the reflection. The intensities of the M3 and M6 reflections were corrected by multiplying by their cross-meridional width (Huxley et al., 1982); the intensities of the other meridional reflections were not width corrected.

The interference components of the M3 and M6 reflections were determined by fitting multiple Gaussian peaks with the same axial width to the meridional intensity distribution, and the total intensity of the reflection was calculated as the sum of the component peaks. The interference components of the M2 reflection were determined by fitting five Gaussian peaks to the meridional intensity distribution; the width of the three lowest angle peaks was constrained to a single value and that of the two highest angle peaks to a different value. The total intensities of the L and H reflections were calculated as the sum of their component peaks. The interference components of the M3 reflection in Fig. 4 C were obtained with a multiple Gaussian fit in which the star peak was fitted with a different axial width from that of the three peaks fitted to the main M3 reflection. The spacing of each reflection was determined from the weighted mean of the component peaks and was calibrated using as a reference the spacing of 14.34 nm (Haselgrove, 1975), measured from the M3 reflection of resting fibers from Rana esculenta at 4°C in Ringer solution, mounted in the same trough as the demembranated rabbit psoas bundles with the same specimen-to-detector distance.

The separations between interference sub-peaks are reported in reciprocal nanometers; a simple 1-D point-diffractor model of the bipolar filament with the first diffractor ∼80 nm from the filament midpoint (the precise value chosen to fit the relative amplitudes of the sub-peaks) was used to estimate the extent and half-filament location of the diffracting units that reproduced the observed separation of the interference sub-peaks. Thus, for example, the sub-peaks of the M3H reflection at 35°C in the presence of Dextran were reproduced with 49 point diffractors in each half filament, with periodicity of 14.36 nm and the first diffractor 80.7 nm from the filament midpoint. The separation between the mid- and high-angle components of the calculated intensity distribution was 1/1,015 nm, close to the experimental value of 1/(1,003 ± 25 nm). These values in nanometers are larger than the physical distance between the centers of the two interfering arrays, 851 nm in this case, because of the convolution of the fringe pattern corresponding to the physical interference distance, with the diffracted intensity distribution from a single array (Huxley et al., 2006; Reconditi, 2006).

To obtain the axial intensity distribution of the first myosin-based layer line (see Fig. 3 A), the 2-D patterns were integrated in the radial region between 0.018 and 0.076 nm^−1^ from the meridional axis (see Fig. 3 B, vertical dashed lines), roughly corresponding to the region between the 1,0 and 1,1 row lines. These integration limits were chosen to collect the major radial peak of the layer line in all conditions studied, and the M1 meridional reflection was almost completely excluded from this radial region. To minimize the contribution from the partially overlapping actin layer line, these 1-D profiles were integrated in the axial region 0.016–0.023 nm^−1^ (see Fig. 3 A, dashed vertical lines) and background-subtracted to obtain the *I*_ML1_ values reported in Fig. 3 C. Black symbols in Fig. 3 D are the centroids of the same background-subtracted profiles in the region 0.016–0.031 nm^−1^. The spacing of the third myosin-based layer line (ML3) was obtained from the centroid of the same axial profiles in the region 0.065–0.075 nm^−1^.

To obtain the radial profile of the first myosin-based layer line (see Fig. 3 B), a series of 243 adjacent vertical strips of three pixels was added and background-subtracted using a convex hull algorithm. The integrated intensity in the axial range 0.014–0.034 nm^−1^ was then plotted against its radial position, and a further background subtraction using a convex hull algorithm was applied to give the profiles in Fig. 3 B.

### Online supplemental material

**Figure S1.**
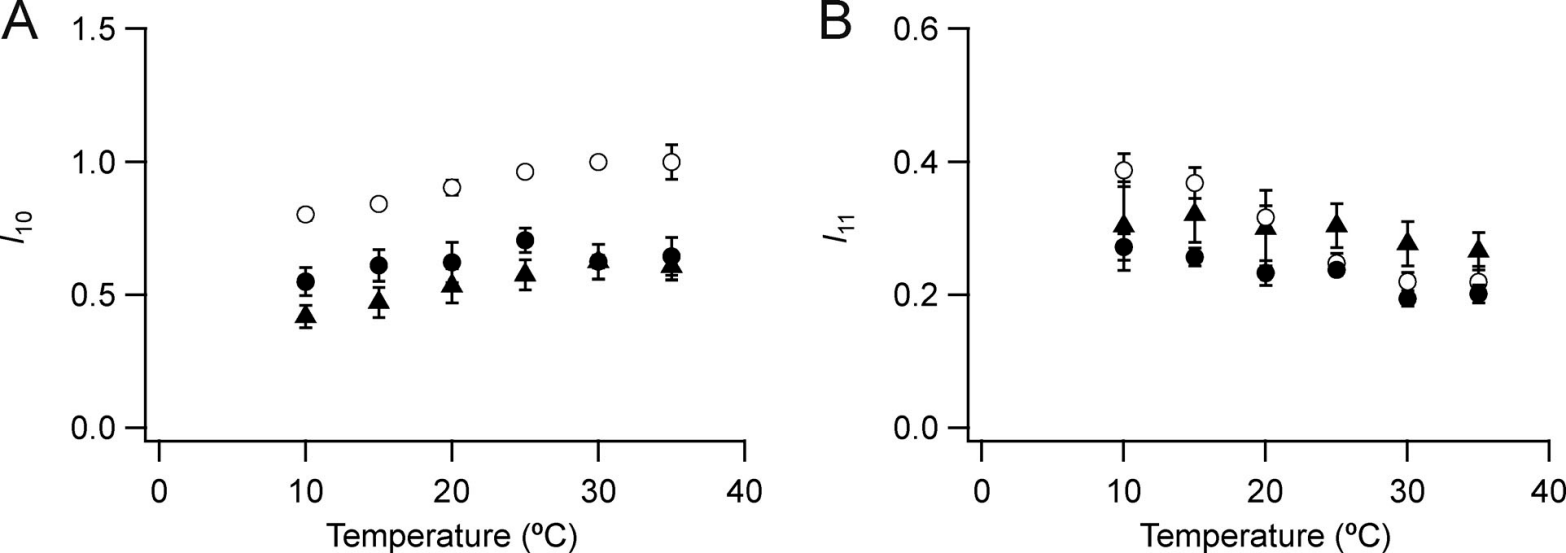
**Intensities of the 1,0 and 1,1 equatorial reflections.** **(A and B)** 1,0 (A) and 1,1 (B) reflections are shown. Mean ± SE. Open circles, no Dextran, *n* = 10 demembranated psoas fiber bundles; filled circles, 5% Dextran, *n* = 6; black triangles, resting intact mouse EDL muscles, *n* = 12, six muscles (Caremani et al., 2019). Intensity of *I*_10_ and *I*_11_ normalized by the value of *I*_10_ at 30°C in the absence of Dextran for the demembranated fibers.

**Figure S2.**
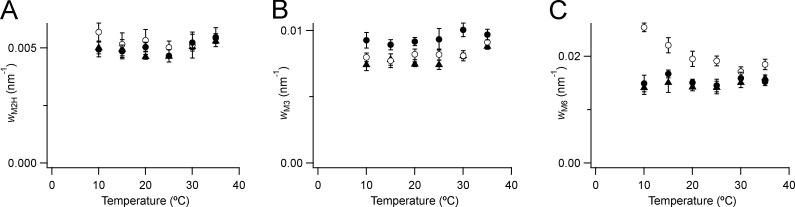
**Radial width of M2, M3, and M6 (C) reflections.** **(A–C)** Data are full width at half-maximum (M2, A; M3, B; and M6, C), mean ± SE. Open circles, demembranated fiber bundles, no Dextran, *n* = 10 (B), 5 (A), and 3 (C). Filled circles, demembranated fiber bundles, 5% Dextran, *n* = 5 (A and B) or 3 (C). Black triangles, resting intact mouse EDL muscles, *n* = 3. *W*, width.

**Figure S3.**
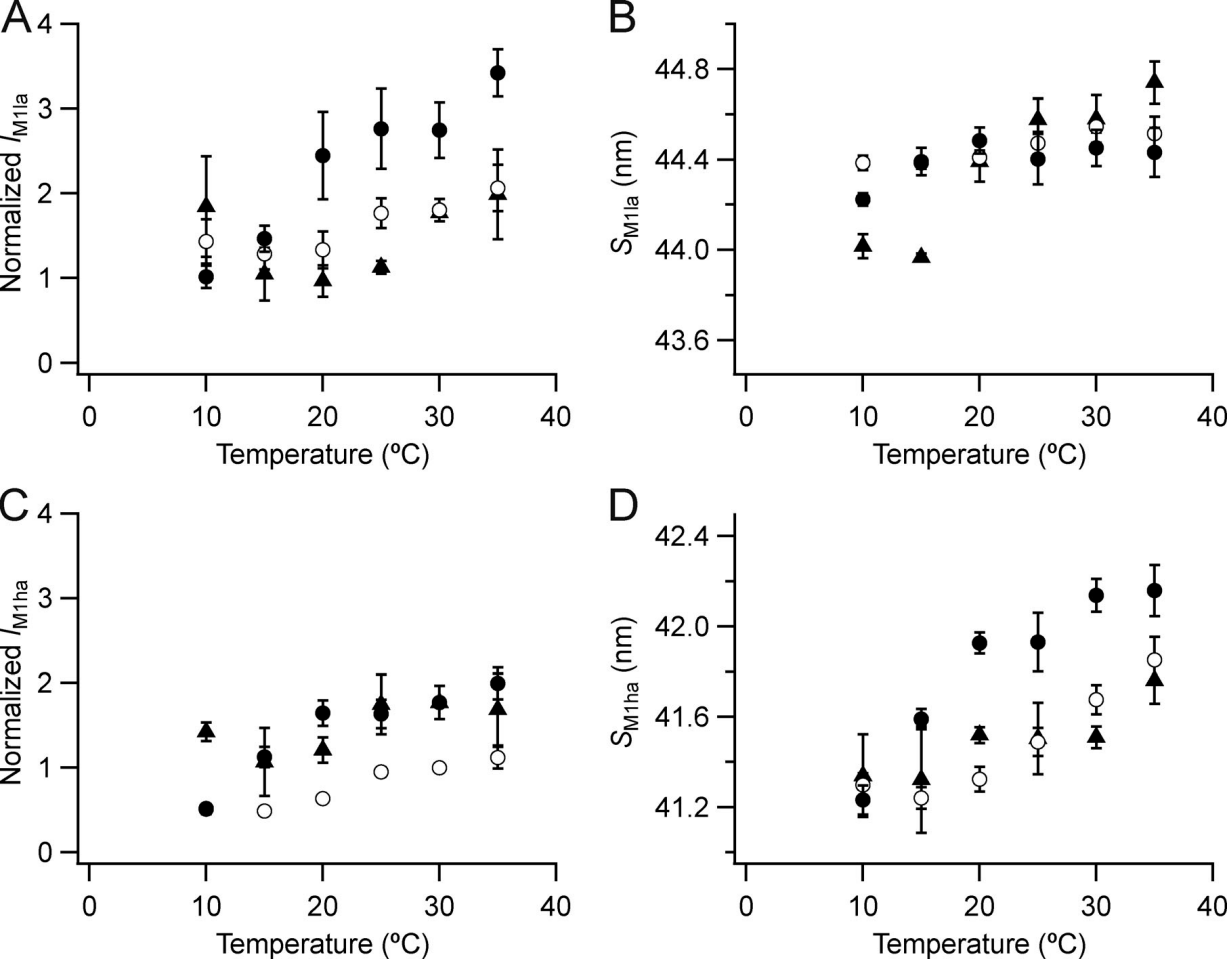
**Intensity and spacing of the component peaks of the M1 reflection.** **(A–D)** Intensity (A and C) and spacing (B and D) are shown. la (A and B) and ha (C and D) denote lower- and higher-angle components. Mean ± SE. Open circles, no Dextran, *n* = 10 demembranated psoas fiber bundles; filled circles, 5% Dextran, *n* = 5; black triangles, resting intact mouse EDL muscles, *n* = 3. Intensities for demembranated fibers normalized by the value of *I*_M1ha_ in the absence of Dextran at 30°C and those for intact muscles scaled to the value for demembranated fibers at 30°C in the presence of Dextran.

**Figure S4.**
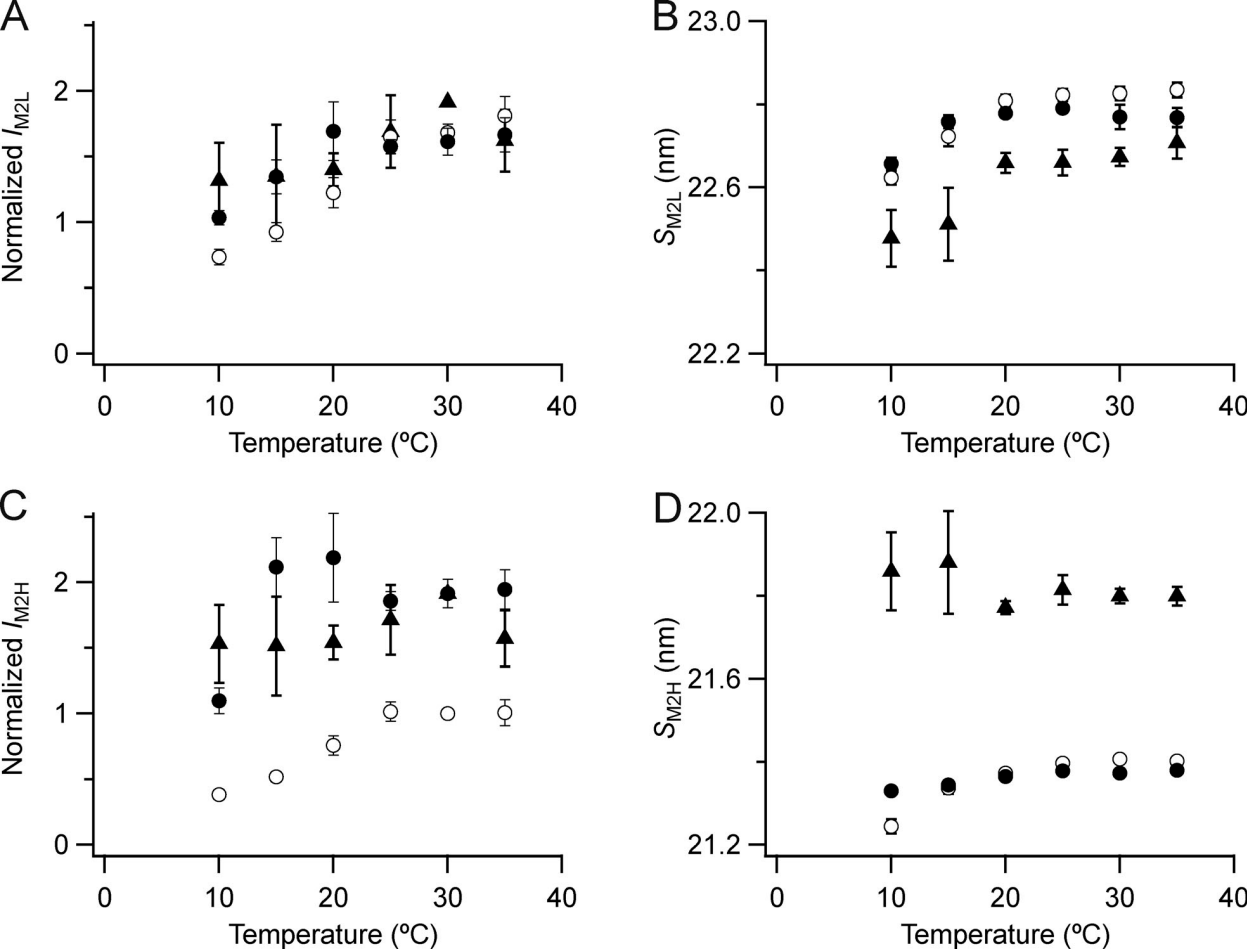
**Intensity and spacing of the component peaks of the M2 reflection.** **(A–D)** Intensity (A and C) and spacing (B and D) are shown. L (A and B) and H (C and D) denote the low- and high-angle pairs, respectively, of sub-peaks in Fig. 4 B. Mean ± SE. Open circles, no Dextran, *n* = 10 demembranated psoas fiber bundles; filled circles, 5% Dextran, *n* = 5; triangles, resting intact mouse EDL muscles, *n* = 3. Intensities for demembranated fibers normalized by *I*_M2H_ in the absence of Dextran at 30°C and those for intact muscles scaled to the value for demembranated fibers at 30°C in the presence of Dextran.

**Figure S5.**
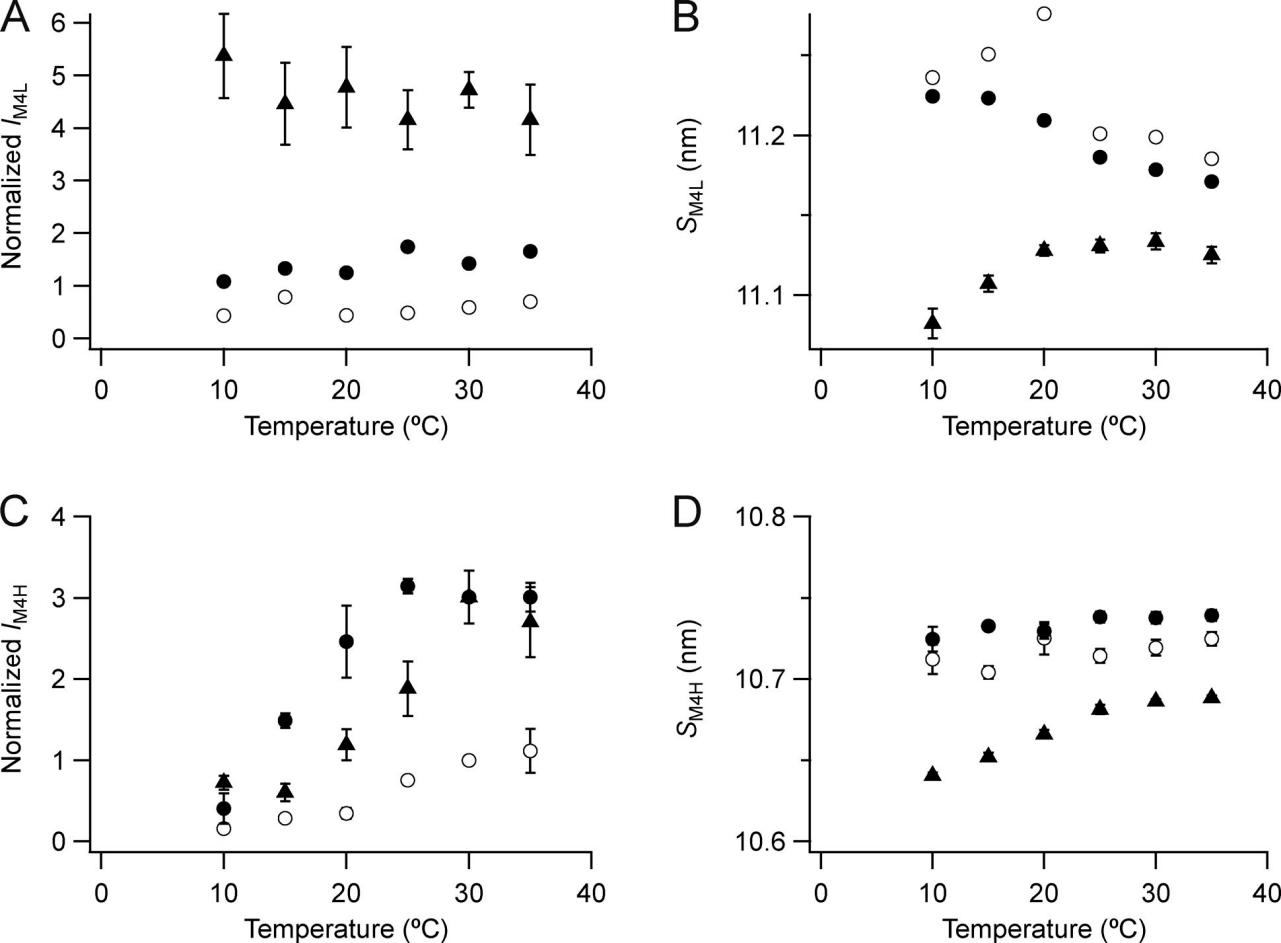
**Temperature dependence of the intensity and spacing of the L and H components of the M4 reflection.** **(A–D)** Intensity (A and C) and spacing (B and D) are shown. L (A and B) and H (C and D) denote the low- and high-angle pairs, respectively. Mean ± SE. Data for M4L added from three bundles; for M4H open circles, no Dextran, *n* = 5 demembranated psoas fiber bundles; filled circles, 5% Dextran, *n* = 3; triangles, resting intact mouse EDL muscles, *n* = 3. Intensities for demembranated fibers normalized by *I*_M4H_ in the absence of Dextran at 30°C and those for intact muscles scaled to the value for demembranated fibers at 30°C in the presence of Dextran.

**Figure S6.**
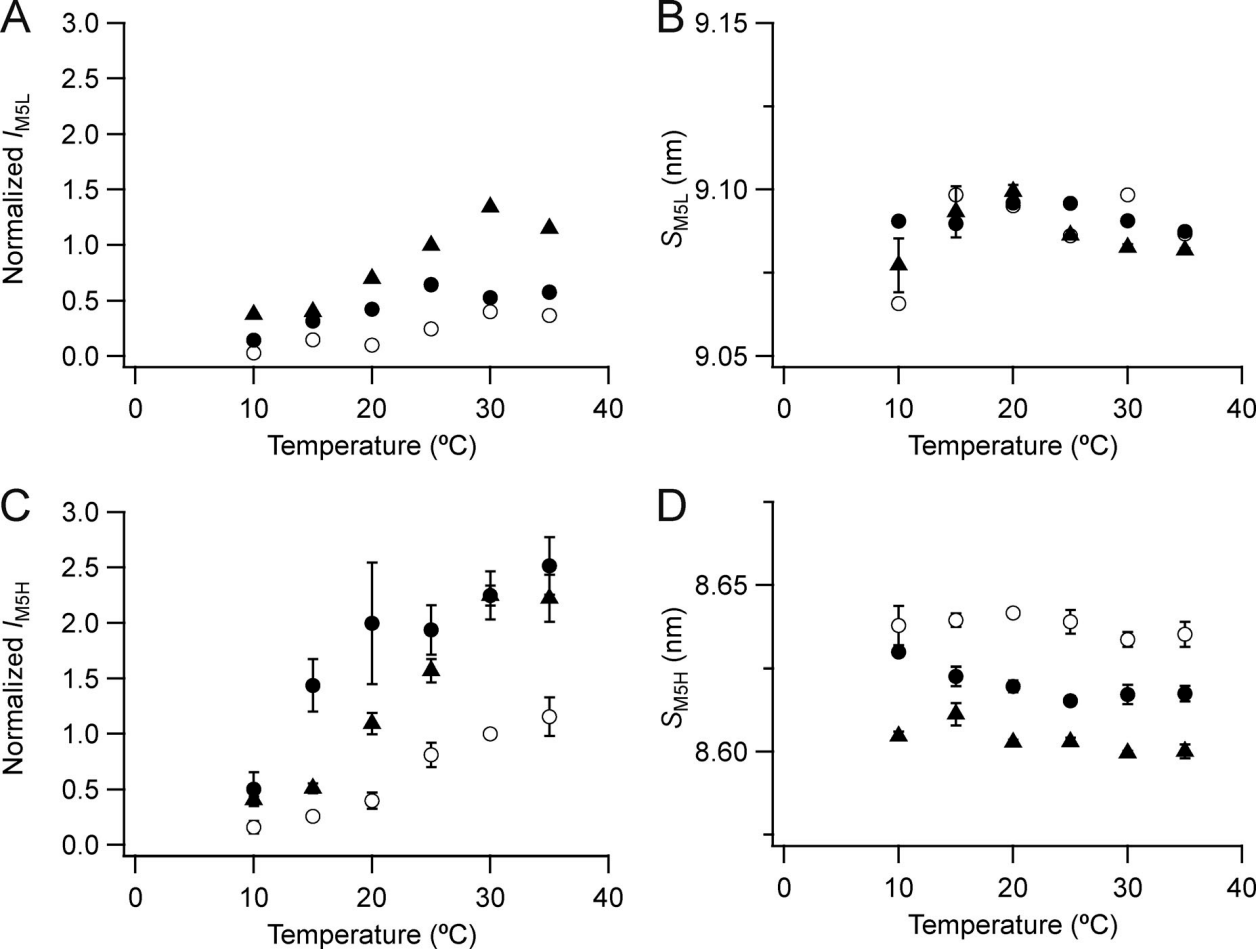
**Intensity and spacing of the L and H components of the M5 reflection.** **(A–D)** Intensity (A and C) and spacing (B and D) are shown. L (A and B) and H (C and D) denote the low- and high-angle pairs, respectively. Mean ± SE. For M5H open circles, no Dextran, *n* = 5 demembranated psoas fiber bundles; filled circles, 5% Dextran, *n* = 3; data for M5L added from three bundles. Black triangles, resting intact mouse EDL muscles, *n* = 3. Intensities for demembranated fibers normalized by *I*_M5H_ in the absence of Dextran at 30°C and those for intact muscles scaled to the value for demembranated fibers at 30°C in the presence of Dextran.

**Figure S7.**
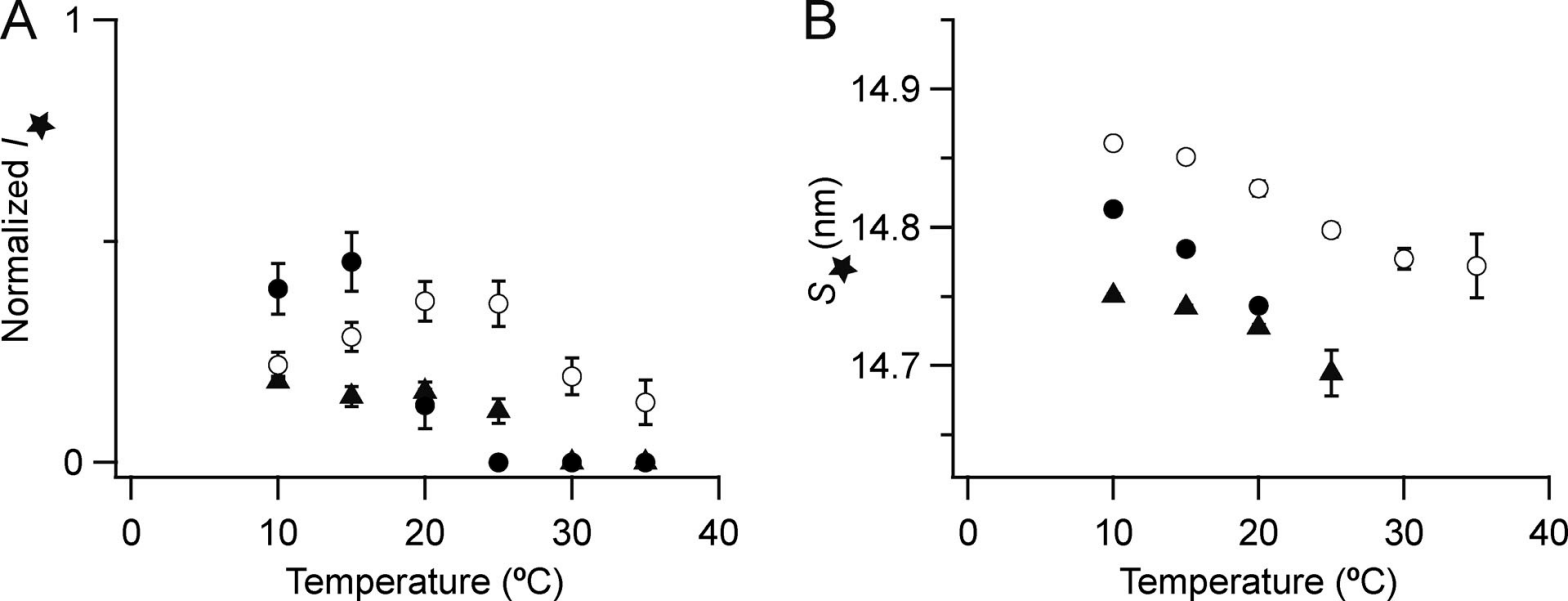
**Intensity and spacing of the star reflection close to the M3 reflection.** **(A and B)** Intensity (A) and spacing (B) are shown. Mean ± SE. Open circles, no Dextran, *n* = 10 demembranated psoas fiber bundles; filled circles, 5% Dextran, *n* = 5; black triangles, resting intact mouse EDL muscles, *n* = 3. Intensities for demembranated fibers normalized by *I*_M3_ in the absence of Dextran at 30°C and those for intact muscles scaled to the value for demembranated fibers at 30°C in the presence of Dextran. The star reflection was not detectable in demembranated fibers at 25–35°C in the presence of Dextran and in intact EDL muscles at 30°C and 35°C; the corresponding intensities are plotted as zeroes in A.

**Figure S8.**
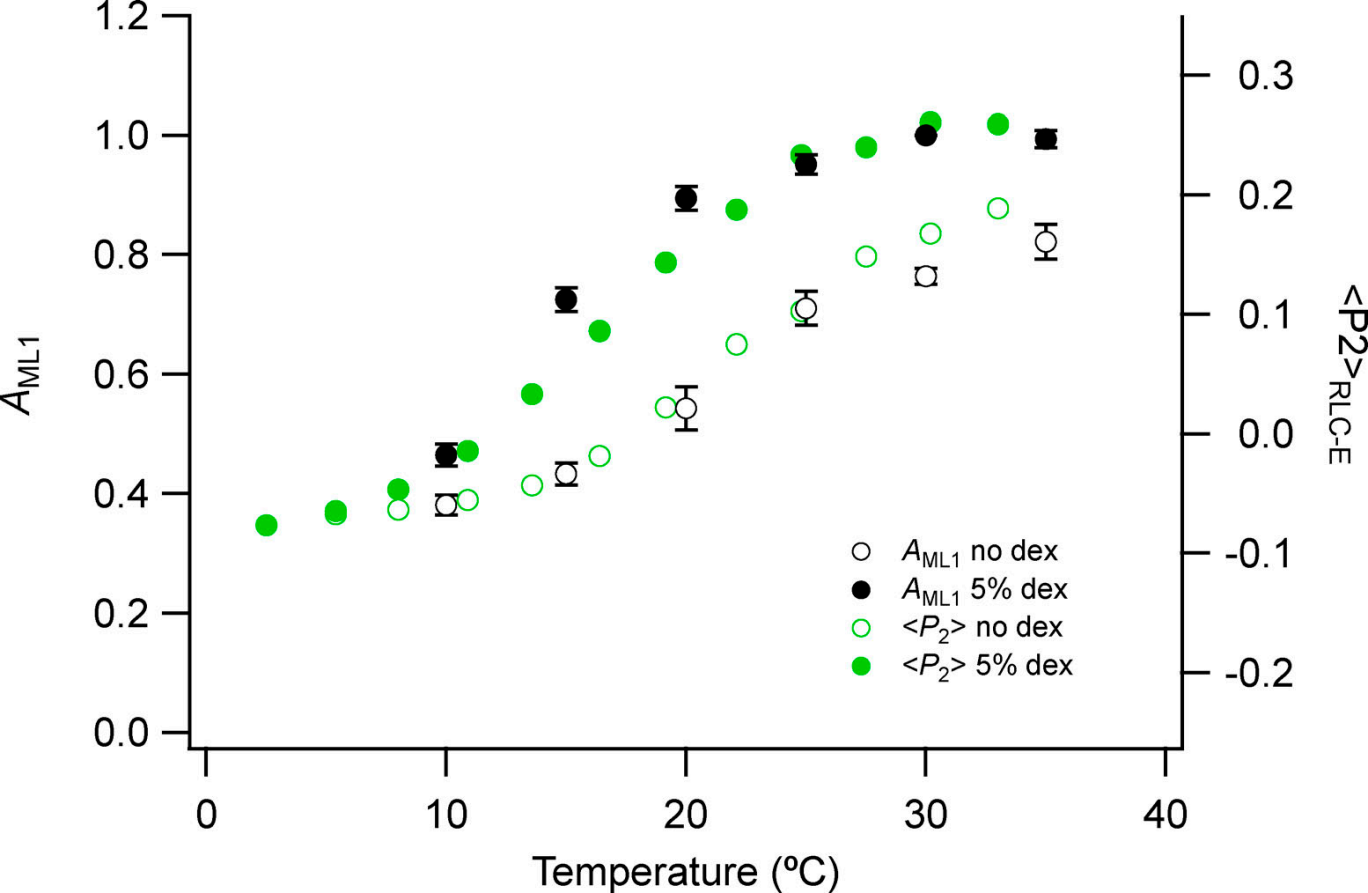
**Fraction of myosin heads in the OFF conformation estimated from the *A*_ML1_ and the orientation parameter <*P*_2_> of a fluorescent probe on the E helix of myosin RLC (RLC-E).** *A*_ML1_ was calculated from the square root of *I*_ML1_ values in Fig 3. Mean ± SE. Open circles, no Dextran, *n* = 10 demembranated psoas fiber bundles; filled circles, 5% Dextran (dex), *n* = 5. Green symbols show <*P*_2_> values from Fig. S5 of Fusi et al. (2015).

**Figure S9.**
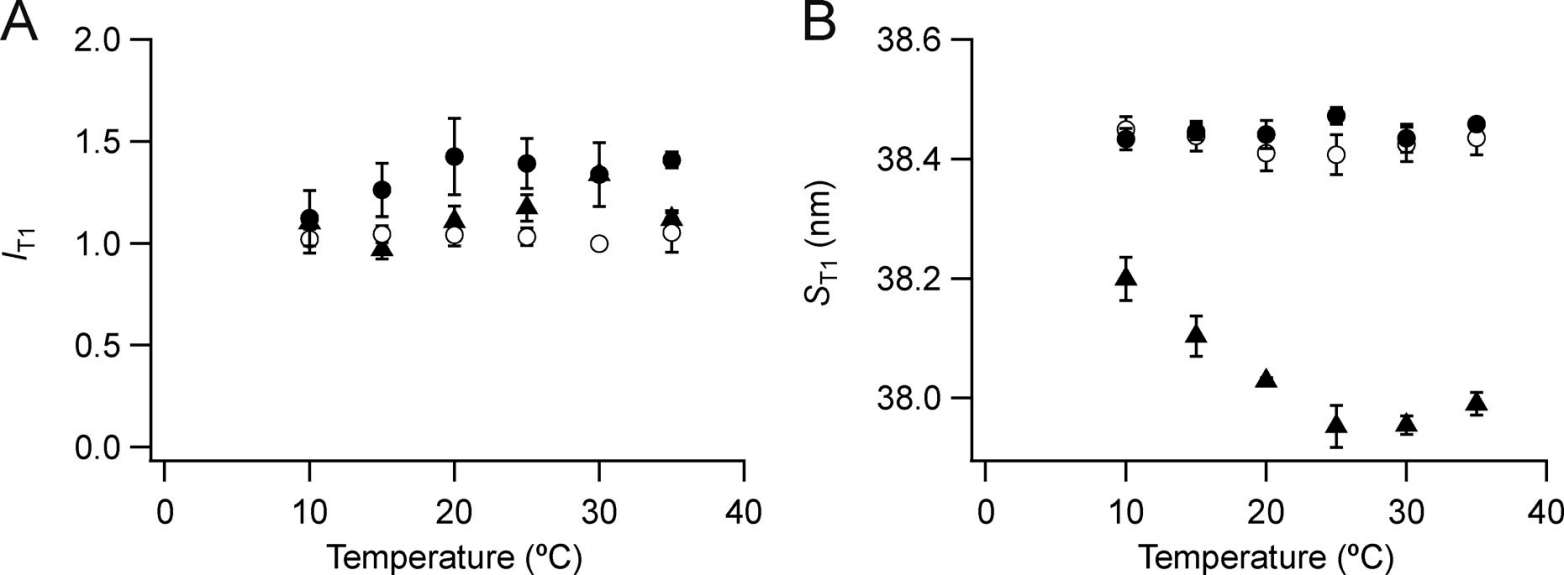
**Intensity and spacing of the T1 reflection.** **(A and B)** Intensity (A) and spacing (B) are shown. Mean ± SE. Open circles, no Dextran, *n* = 10 demembranated psoas fiber bundles; filled circles, 5% Dextran, *n* = 5; triangles, resting intact mouse EDL muscles, *n* = 3. Intensities are normalized by the value at 30°C in the absence of Dextran.

**Figure S10.**
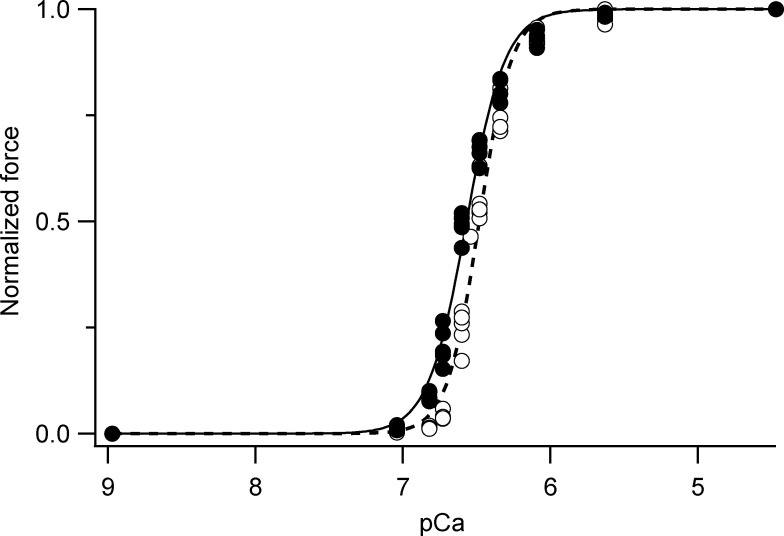
**Force-pCa titrations.** Steady-state force at different pCa (= −log_10_[Ca^2+^]) normalized by the maximum force at pCa 4.7 in the presence (filled circles) and in the absence (open circles) of 5% Dextran. Data pooled from T-jump experiments from 1–12°C on single demembranated fibers from psoas muscle (*n* = 5 fibers). Thin continuous (dashed) line is Hill-curve fitted to the data in the presence (absence) of 5% Dextran with fitting parameters pCa_50_ = 6.48 ± 0.01 (6.57 ± 0.01) and *n* = 3.94 ± 0.21 (3.30 ± 0.14), mean ± std from the fitting.

**Figure S11.**
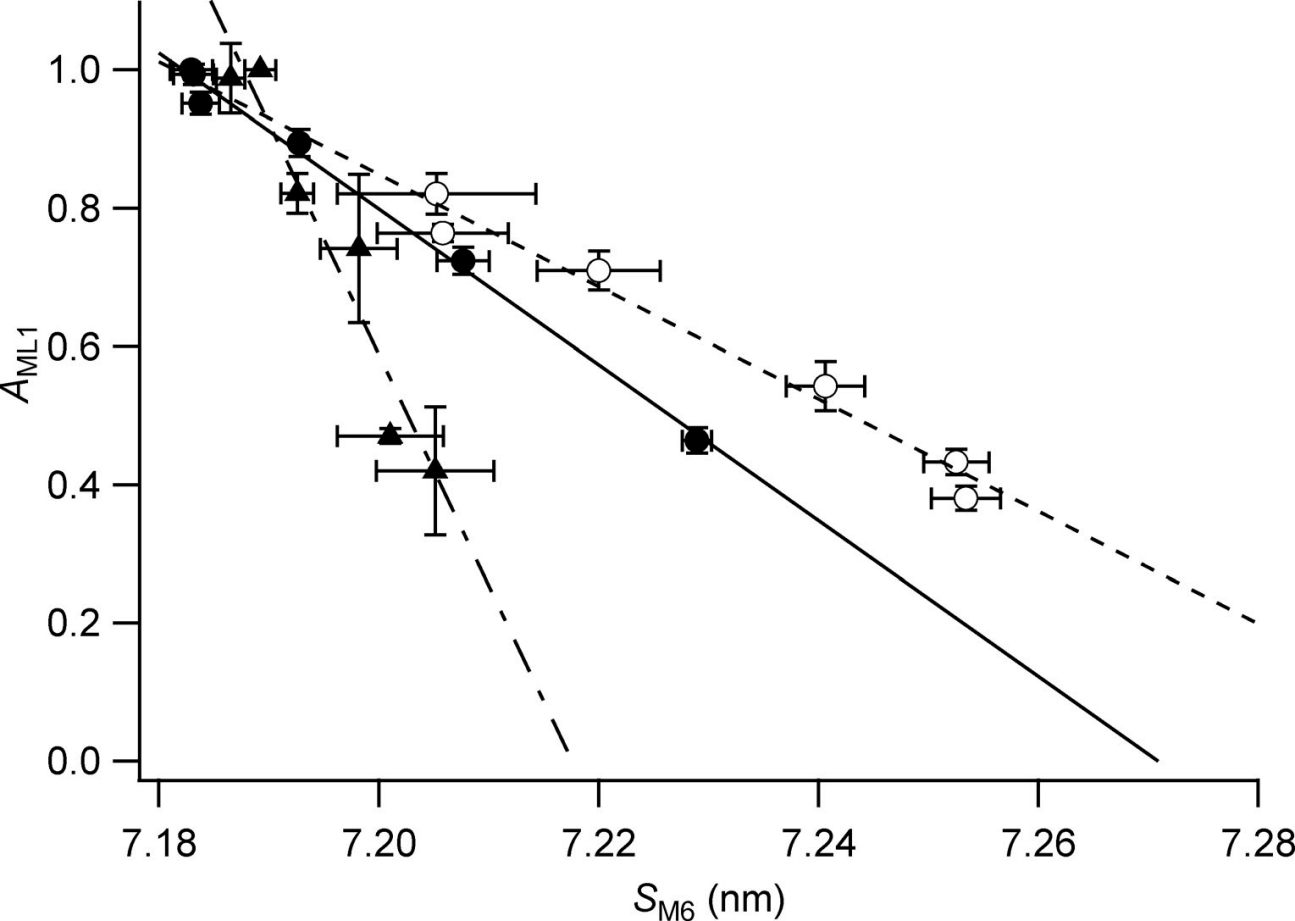
**Relationship between the relative amplitude of the first myosin layer line (*A*_ML1_) and the myosin filament backbone spacing (*S*_M6_).** Amplitude of the first myosin layer line calculated as *A*_ML1_ (the square root of *I*_ML1_) from the data in Fig. 3 C for demembranated fibers in the absence (open circles) or presence (filled circles) of Dextran and for intact muscles (triangles). The *S*_M6_ data are the same as in Fig. 5 B. Error bars denote SE. The lines indicate linear regression for each set of data.

Fig. S1 reports the temperature dependence of the intensity of the equatorial reflections 1,0 and 1,1 for demembranated psoas muscle fiber bundles and intact EDL muscle. Fig. S2 shows the temperature dependence of the radial width of the M2, M3, and M6 meridional reflections. Fig. S3, Fig. S4, Fig. S5, and Fig. S6 show the temperature dependence of the intensity and spacing of the low- and high-angle peaks of the M1, M2, M4, and M5 meridional reflections, respectively. Fig. S7 shows the temperature dependence of the intensity and spacing of the meridional star reflection close to the M3 reflection. Fig. S8 compares the fraction of myosin heads in the OFF conformation estimated from the relative amplitude of the first myosin layer line (*A*_ML1_) and the orientation parameter <*P*_2_> of fluorescent probe on the myosin regulatory light chain (RLC). Fig. S9 shows the temperature dependence of the intensity and spacing of the T1 reflection. Fig. S10 shows the force-pCa titrations in isolated psoas muscle fibers or bundles at 12°C in the presence and absence of Dextran. Fig. S11 compares the relationship between *A*_ML1_ and the myosin filament backbone spacing (*S*_M6_) of demembranated psoas muscle fibers and intact EDL muscle. Table S1 reports the spacing of the component peaks of the M2, M3, and M6 meridional reflections at 35°C in demembranated bundles of psoas muscle fibers in the presence of 5% Dextran T500 and in intact EDL muscle at rest.

## Results

### Small-angle x-ray diffraction from relaxed mammalian muscle

**Figure 1.**
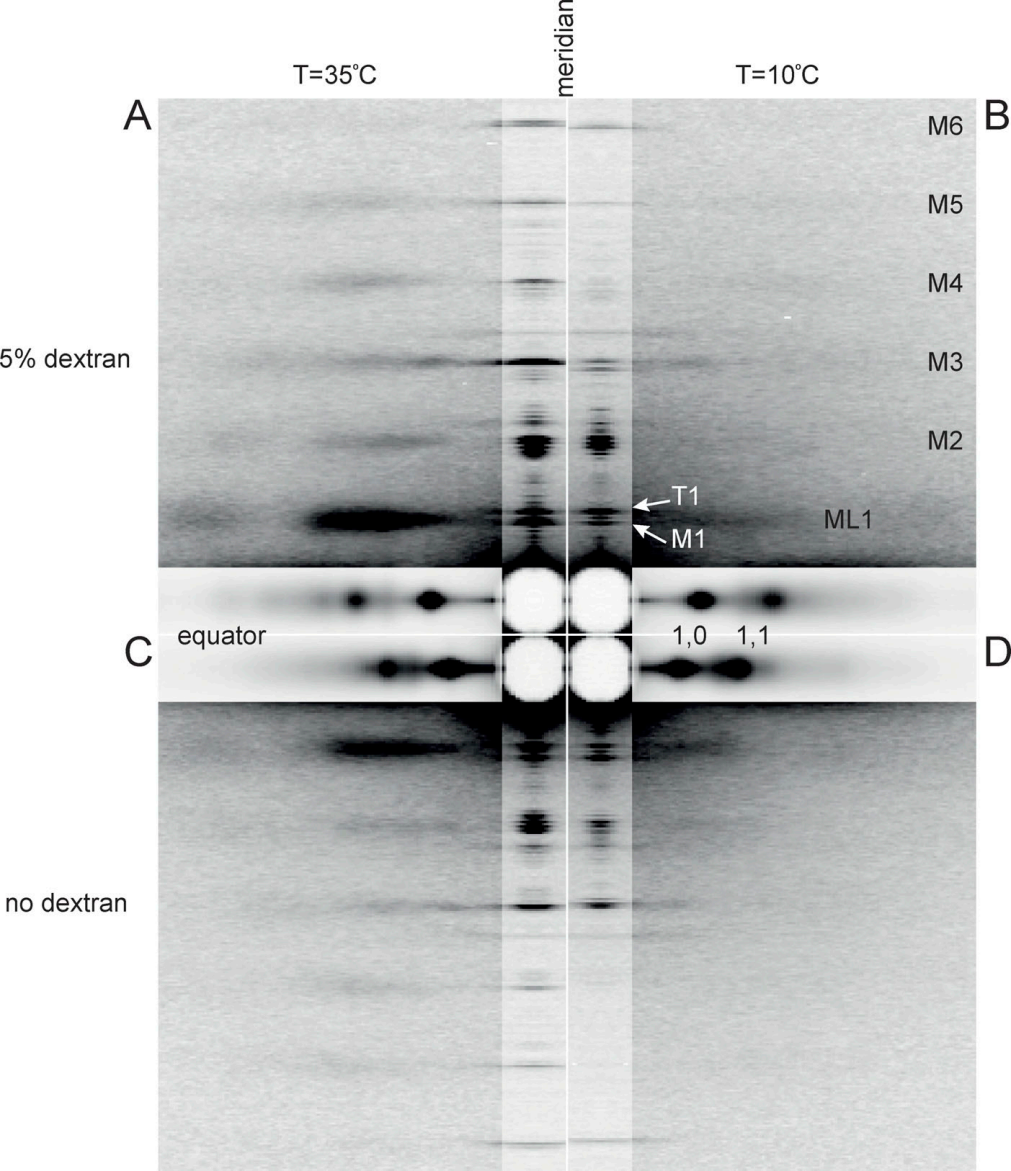
**Low-angle x-ray diffraction patterns from demembranated fibers from rabbit psoas muscle in relaxing conditions.** **(A–D)** Patterns at 35°C (A and C) and at 10°C (B and D), in the presence (A and B) or absence (C and D) of 5% Dextran T500. The meridional and equatorial axes (parallel and perpendicular to the fiber axis) were digitally attenuated 2 and 20 times, respectively. Data added from three fiber bundles; total exposure time, 60 ms; specimen-to-detector distance, 3 m. Mi, myosin-based reflections indexing on its ∼43-nm axial repeat; ML1, first myosin layer line; T1, troponin-based reflection with axial periodicity ∼38 nm.

Bundles of 10–15 fibers, 5–6 mm long, were dissected from demembranated rabbit psoas muscle and mounted horizontally at sarcomere length 2.5 µm in relaxing solution at beamline ID02 of the ESRF (see Materials and methods). For comparison, intact EDL muscles of the mouse were mounted vertically in physiological saline at sarcomere length ∼2.6 µm at the BioCAT beamline (APS; Caremani et al., 2019). Small-angle x-ray diffraction patterns recorded with high spatial resolution and S/N at these beamlines (Fig. 1; Caremani et al., 2019) exhibit the well-known features of the muscle x-ray diagram. The two most prominent equatorial reflections are designated 1,0 and 1,1 in reference to the corresponding planes of the hexagonal lattice of the thick and thin filaments. The most prominent meridional reflections, designated M1 to M6, are usually considered to be orders of the fundamental myosin-based spatial periodicity of ∼43 nm corresponding to the M1 reflection, although each of these meridional reflections has multiple components. A second series of meridional reflections, the “T” series, indexes on ∼38 nm (T1), corresponding to the axial periodicity of troponin in the thin filaments. The broad off-meridional layer lines associated with each M reflection (ML1, etc.) signal the quasi-helical organization of the myosin head or motor domains on the surface of the thick filament in the relaxed, resting, or OFF state.

All these x-ray reflections are sensitive to temperature in the range of 35°C (Fig. 1, A and C) to 10°C (Fig. 1, B and D) and in demembranated muscle fibers to lattice compression by the addition of 5% Dextran to the relaxing solution (Fig. 1, A and B) compared with the absence of Dextran (Fig. 1, C and D). Lattice compression by Dextran is indicated by the movement of the equatorial 1,0 and 1,1 reflections away from the center of the pattern but also produces characteristic changes in their intensities. The intensities of the meridional and layer-line reflections are generally increased by the addition of Dextran, but are more substantially decreased by cooling from 35°C to 10°C. The spacings of these reflections, related to axial periodicities in the thick filament, and the splitting of each reflection into sub-peaks, related to the conformation of the myosin head domains (Linari et al., 2000; Reconditi et al., 2011), are also sensitive to temperature and to lattice compression, as described below.

### Equatorial reflections

**Figure 2.**
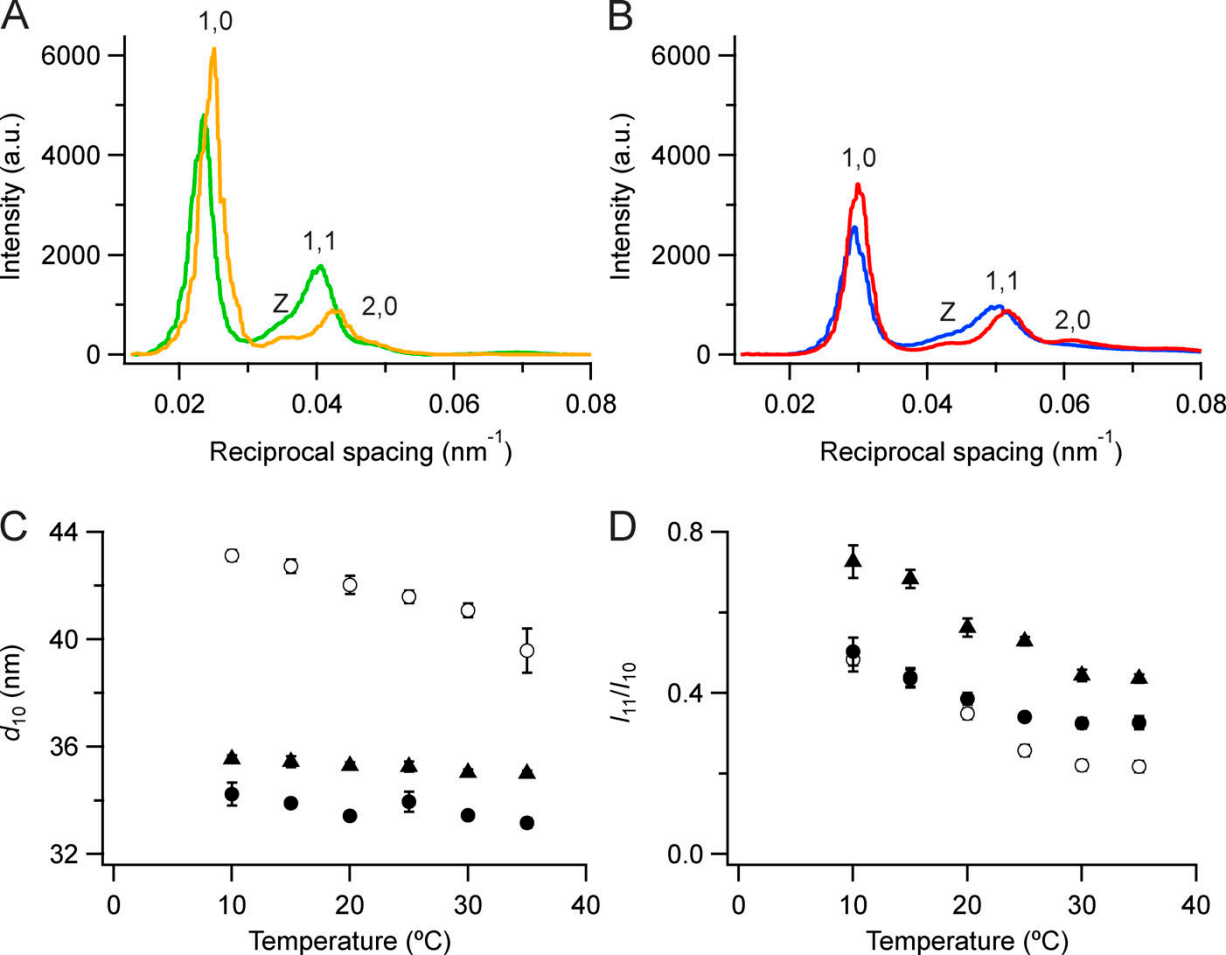
**Equatorial x-ray reflections.** **(A and B)** Intensity distributions along the equator at 35°C (orange, red) and 10°C (green, blue) in demembranated muscle fibers in the absence (A) and presence (B) of 5% Dextran, from the diffraction patterns in Fig. 1. **(C)** Spacing of the 1,0 reflection (*d*_10_). **(D)** Ratio of the intensities of the 1,1 and 1,0 reflections (*I*_11_/*I*_10_); mean ± SE. Open circles, no Dextran, *n* = 10 demembranated fiber bundles; filled circles, 5% Dextran, *n* = 6. Black triangles in C and D are from resting intact mouse EDL muscles (*n* = 12, six muscles; Caremani et al., 2019). a.u., arbitrary units.

The equatorial 1,0 and 1,1 reflections can be characterized by the distribution of diffracted intensity along the equator of the x-ray diagram (Fig. 2, A and B). The 1,0 reflection is more intense than the 1,1 in relaxed demembranated and resting intact muscle (Haselgrove and Huxley, 1973), indicating that the myosin motors are close to the surface of the thick filaments, which are centered on the 1,0 planes of the filament lattice. This difference is more pronounced at higher temperatures (Fig. 2 A, orange; and Fig. 2 B, red). The 1,0 reflection is weaker in the presence of Dextran (Fig. 2 B) than in its absence (Fig. 2 A).

The intensities and spacings of the 1,0 and 1,1 reflections were determined by fitting the equatorial intensity distributions with multiple Gaussian peaks, corresponding to the 1,0, 1,1, and 2,0 reflections from the region of the sarcomere in which thick and thin filaments overlap and the “Z” reflection from the lattice of thin filaments at the Z-band (Fig. 2, A and B; Millman, 1998). The spacing of the 1,0 reflection, *d*_10_, was much lower in the presence of 5% Dextran (Fig. 2 C, filled circles) than in its absence (open circles), as expected from osmotic compression of the filament lattice, and was significantly lower than values measured in the same temperature range in mouse EDL muscles at rest (triangles; Caremani et al., 2019). *d*_10_ in the absence of Dextran monotonically increases as temperature is lowered, and this effect was strongly attenuated in the presence of Dextran and in resting intact muscles. The effects of varying temperature in the latter two conditions occur at almost constant interfilament separation.

The ratio of the intensities of the 1,1 and 1,0 reflections, *I*_11_/*I*_10_ (Fig. 2 D), is generally regarded as a signal for the movement of myosin motors away from the thick filament backbone toward the thin filaments. *I*_11_/*I*_10_ is 0.32 in demembranated fibers from rabbit psoas muscle at 35°C in the presence of Dextran and 0.44 in intact mouse EDL muscles at the same temperature at rest (Fig. 2 D). It increases to almost 2.0 during active contraction of psoas bundles at 30°C (Ferenczi et al., 2005) or mouse EDL at 35°C (Caremani et al., 2019). *I*_11_/*I*_10_ increases by a smaller extent on cooling in both relaxed and resting muscle (Fig. 2 D; Xu et al., 1997; Caremani et al., 2019) and is slightly larger in the presence of Dextran at 35°C but not at 10°C. It is also larger in resting mouse EDL muscle (triangles) than in demembranated rabbit psoas muscle at each temperature, even in the presence of Dextran. Cooling decreases *I*_10_ and increases *I*_11_ in demembranated fibers in the absence of Dextran, but the changes are smaller in the presence of Dextran and in intact resting muscle (Fig. S1).

### Myosin-based layer lines

**Figure 3.**
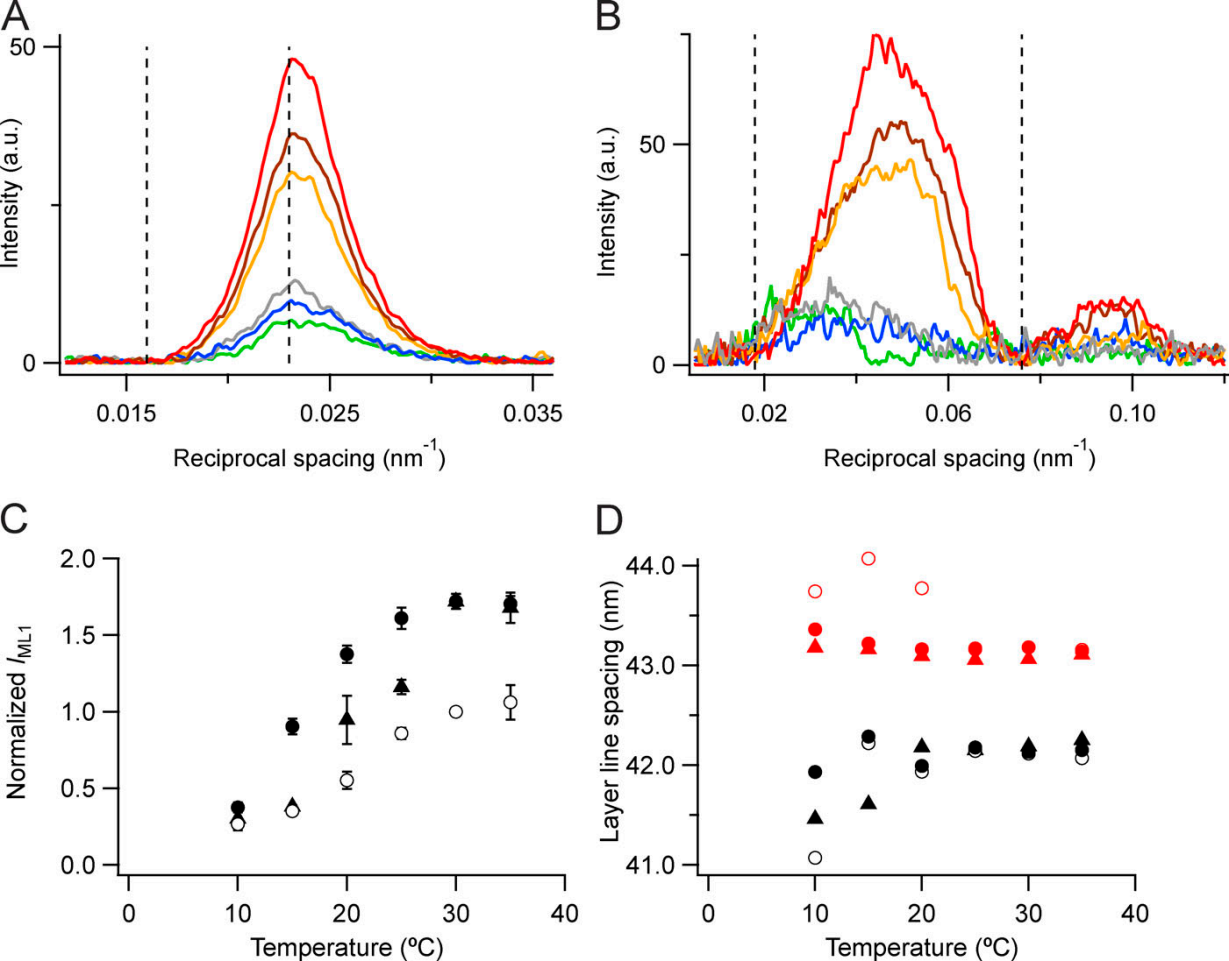
**Layer-line reflections.** **(A and B)** Axial (parallel to the meridian; A) and radial (perpendicular to the meridian; B) intensity distributions of the first layer line in demembranated muscle fibers. With Dextran: red, 35°C; brown, 20°C; and blue, 10°C. Without Dextran: orange, 35°C; gray, 20°C; and green, 10°C. Vertical dashed lines in A indicate integration limits used to calculate *I*_ML1_; those in B indicate integration limits used to calculate the meridional profiles in A. **(C)** Intensity of the first myosin layer line (*I*_ML1_) normalized for demembranated fibers (filled circles) by the value in the absence of Dextran at 30°C (open circles) and for intact muscles (triangles; Caremani et al., 2019) by scaling to the value for demembranated fibers at 30°C in the presence of Dextran (filled circles). Error bars and corresponding *n* values as in Fig. 2 C. **(D)** Spacing of the mixed layer line (*S*_LL1_) with symbols as in C and the fundamental myosin-based helical periodicity estimated as 3 * *S*_LL3_ (corresponding red symbols). Data added from five bundles of demembranated fibers and from three intact EDL muscles to increase S/N. See text for details. a.u., arbitrary units.

The myosin-based layer lines (ML1 and its higher orders; Fig. 1), signaling the helical order of the myosin motors on the surface of the thick filaments, are much stronger at higher temperature, and at high temperature are stronger in the presence of Dextran (Fig. 3). The first myosin-based layer line (ML1) partially overlaps the first actin-based layer line (AL1), and the ML1 intensity (*I*_ML1_) was estimated from a selected region of the layer line in order to minimize the contribution of AL1 (see Materials and methods). *I*_ML1_ has an approximately sigmoidal dependence on temperature (Fig. 3 C), with a transition temperature, estimated as the temperature at which the change in *I*_ML1_ is half-complete, of ∼22°C in demembranated fibers in the absence of Dextran (open circles) and 16°C in its presence (filled circles). The temperature-dependence of *I*_ML1_ in demembranated fibers from rabbit psoas muscle is broadly similar to that in resting intact EDL muscle of the mouse (triangles).

The axial spacing of the mixed first layer line (*S*_LL1_), determined from a Gaussian fit of the axial profiles in Fig. 3 A, was 42.2 nm at 35°C independent of the presence of Dextran (Fig. 3 D, filled black circles), almost the same as the value for resting mouse EDL muscle at 35°C (42.3 nm; black triangles). These values are intermediate between the canonical periodicities of the myosin-based and actin-based helices, ∼43 and 38 nm, respectively (Huxley and Brown, 1967). Since *S*_ML1_ cannot be determined accurately from the axial profile of the first layer line, we used the axial profile of the third-order myosin-based layer line (ML3), which is well separated from any actin-based contribution, for this purpose. The axial spacing of the ML3 layer line (*S*_ML3_) was 14.39 nm at 35°C, independent of the presence of Dextran, corresponding to an *S*_ML1_ of 43.17 nm (calculated as 3 * *S*_ML3_; Fig. 3 D, red circles; and Table 1). *S*_ML1_ determined by the same method in intact resting mouse EDL was almost identical, 43.11 nm (Table 1). *S*_ML1_ is almost independent of temperature at 20°C and above in both demembranated and intact muscle, but it increases at lower temperatures in demembranated fibers in the absence of Dextran (open red circles).

**Table 1.** Spacings in nm of the myosin-based layer line and meridional reflections at 35°C in demembranated bundles of psoas muscle fibers in the presence of 5% Dextran T500 and in intact EDL muscle at rest

		Demembranated relax			Intact resting	
		Mean	SE	Expected L and H	Mean	SE
L fundamental				45.537		
ML1		43.170		43.140	43.110	
M1	la	44.432	0.109	45.537	44.740	0.093
	ha	42.159	0.011	43.140	41.762	0.103
M2	L	22.769	0.023	22.769	22.707	0.039
	H	21.380	0.006	21.570	21.799	0.023
M3	L			15.179		
	H	14.372	0.004	14.380	14.347	0.002
M4	L	11.171		11.384	11.125	0.005
	H	10.739	0.003	10.785	10.688	0.002
M5	L	9.087		9.107	9.082	0.001
	H	8.617	0.002	8.628	8.593	0.001
M6	L			7.590		
	H	7.183	0.002	7.190	7.187	0.001

SE, standard error.

The radial profiles of the myosin-based layer lines give information about the radial mass distribution of the myosin motors with respect to the thick filament backbone. The radial profile of the first layer line (Fig. 3 B) has a prominent inner peak with a reciprocal spacing of ∼1/22 nm at 35°C, roughly independent of the presence of Dextran, and similar to the corresponding profile from resting EDL muscle of the mouse (Fig. S3 of Caremani et al., 2019) in which the corresponding radial peak is at ∼1/20 nm. These peaks move closer to the meridian at lower temperatures, indicating that the myosin head domains move farther from the thick filament surface.

### Myosin-based meridional reflections

**Figure 4.**
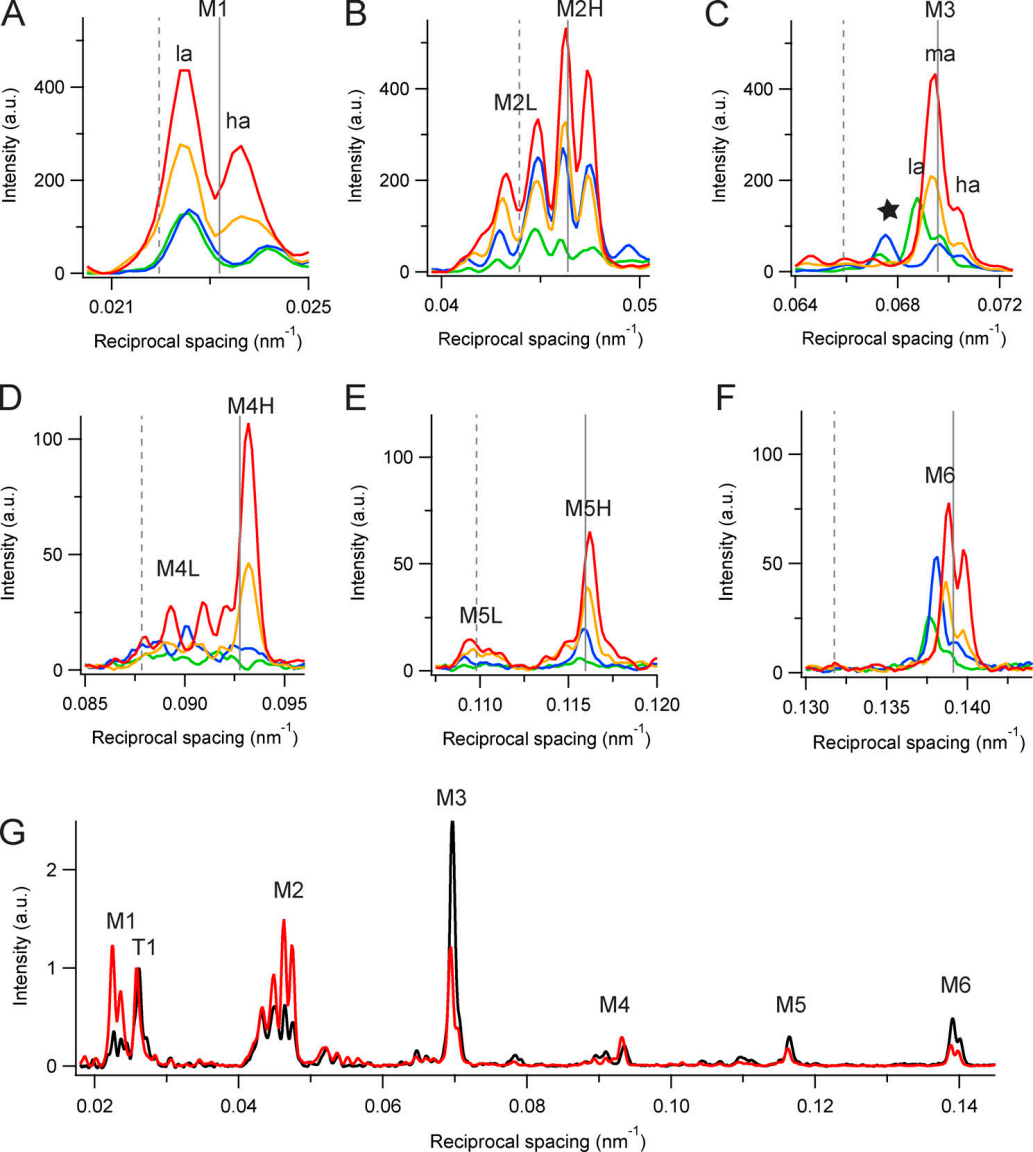
**Meridional intensity distributions.** **(A–F)** Myosin-based reflections M1 (A), M2 (B), M3 (C), M4 (D), M5 (E), and M6 (F) at 35°C (red and orange) and 10°C (blue and green) in the presence (red and blue) and in the absence (orange and green) of 5% Dextran in demembranated muscle fibers, from the data in Fig. 1. Vertical dashed and continuous gray lines indicate multiples of the fundamental L and H periodicities, respectively. **(G)** Meridional intensity distributions at 35°C from demembranated fibers in the presence of Dextran (red, same data as panels A–F) and from intact EDL muscle of the mouse (black, Caremani et al., 2019), normalized by the main peak of the T1 reflection. a.u., arbitrary units.

The myosin-based meridional reflections M1 to M6 (Fig. 1) were characterized using the diffracted intensity distributions along the meridional axis (Fig. 4). Each M reflection is split into a closely spaced set of sub-peaks. In general, the M reflection sub-peaks are stronger at higher temperature and in the presence of Dextran (Fig. 4, A–F). The meridional intensity distribution from relaxed demembranated fibers at 35°C in the presence of Dextran (Fig. 4 G, red) is similar in many respects to that from intact EDL muscles of the mouse at the same temperature (black), with one major difference: the M1 and M2 reflections are stronger in the demembranated preparation, but the M3 and M6 are weaker. These differences are not due to systematic differences in the radial widths of the reflections, which are similar in demembranated fibers from rabbit psoas muscle and intact resting mouse EDL muscles (Fig. S2).

#### The M6 reflection

The M6 reflection, with a spacing of ∼7.2 nm, is present in both resting muscle and during active contraction, but it is relatively insensitive to mechanical perturbations in active muscle, suggesting that it is dominated by diffraction by a structural periodicity in the thick filament backbone, with only a minor contribution from the myosin head domains (Reconditi et al., 2004; Huxley et al., 2006). The spacing of the M6 reflection, *S*_M6_, increases by ∼1.5% on activation; the thick filament is slightly longer in its ON state (Huxley and Brown, 1967; Haselgrove, 1975). *S*_M6_ has therefore been used as a signal for the regulatory state of the thick filament backbone.

The M6 reflection (Fig. 4 F) has two clear sub-peaks due to x-ray interference between the diffracting structures in the two halves of the bipolar filament backbone (Linari et al., 2000; Juanhuix et al., 2001; Huxley et al., 2006). The sub-peak spacings at 35°C in the presence of Dextran are reported in Table 1. *S*_M6_, determined as the weighted mean of the two sub-peaks, is 7.183 nm in these conditions, corresponding to the sixth order of a fundamental myosin-based periodicity of 43.10 nm, matching that of the myosin-based helix determined in the previous section (Table 1). Essentially the same value was measured in resting intact mouse EDL muscle at the same temperature (Table 1).

**Figure 5.**
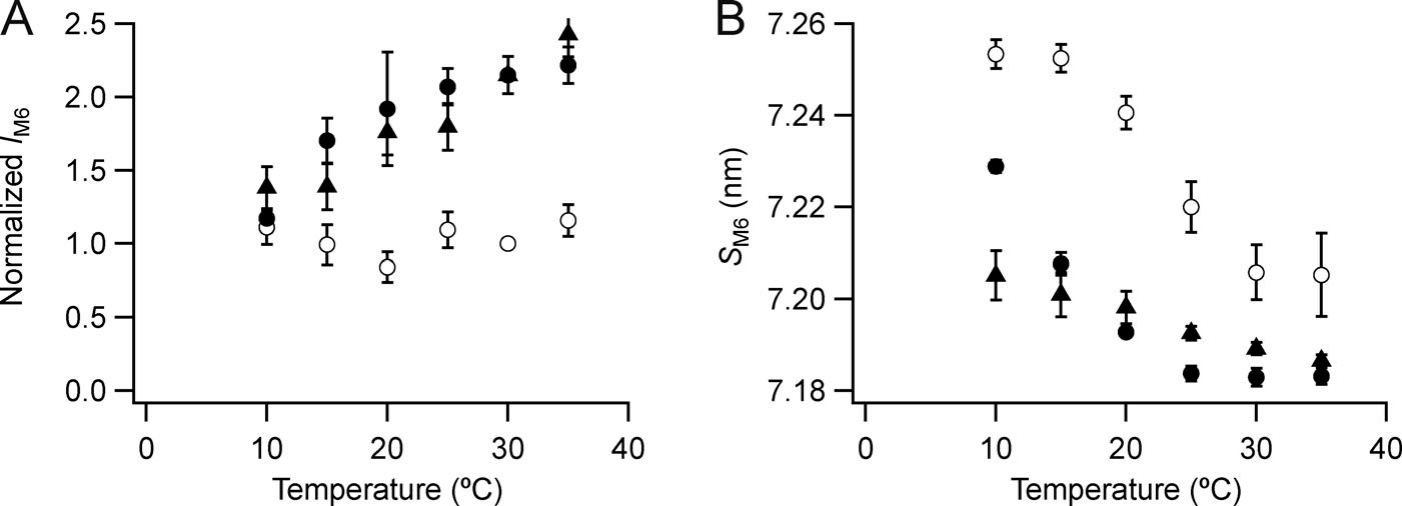
**Intensity and spacing of the M6 reflection.** **(A and B)** Intensity (A) and spacing (B) are shown. Mean ± SE. Open circles, no Dextran, *n* = 5 demembranated psoas fiber bundles; filled circles, 5% Dextran, *n* = 3; black triangles, resting intact mouse EDL muscles, *n* = 6. Intensities for demembranated fibers normalized by the value in the absence of Dextran at 30°C and those for intact muscles scaled to the value for demembranated fibers at 30°C in the presence of Dextran.

The M6 reflection is stronger at higher temperatures and in the presence of Dextran (Fig. 4 F). Its intensity (*I*_M6_; Fig. 5 A) is almost independent of temperature in the absence of Dextran (open circles), but it decreases on cooling in its presence (filled circles). *I*_M6_ shows a similar decrease on cooling in resting intact EDL muscle (triangles). The spacing of the M6 reflection (*S*_M6_; Fig. 5 B) increased by 0.6% on cooling from 35°C to 10°C in the presence of Dextran (filled circles) and by 0.7% in its absence (open circles). The increase on cooling of resting intact muscle was smaller, 0.25% (triangles). In demembranated fibers, *S*_M6_ is higher in the absence of Dextran at all temperatures studied, and lattice compression at temperatures >10°C reduces its value to close to that observed in intact muscle. The transition temperature for the change in *S*_M6_ in the absence of Dextran, 23°C, is close to that for *I*_ML1_, and the relationship between each structural parameter and temperature is shifted to lower temperature in the presence of Dextran.

#### The “forbidden” reflections: M1, M2, M4, and M5

If the arrangement of the myosin heads on the surface of the thick filament followed a perfect three-start helix, the meridional reflections M3 and M6 (and M9, M12, etc.) would be strong, but reflections of orders that are not multiples of three would be absent. The latter are therefore referred to as forbidden reflections, and their presence suggests that the helical arrangement of the heads is systematically perturbed within each fundamental 43-nm repeat, although it is also possible that there is a contribution from the higher-order axial reflections from another thick filament component with the same axial periodicity that does not follow a three-start helix.

The M1 reflection appears as two main sub-peaks (Fig. 4 A), and the lower-angle sub-peak (M1_la_) is more intense than the higher-angle sub-peak (M1_ha_), although in resting intact muscle M1_ha_ itself may be split (Fig. 4 G, black). The intensities of the two main sub-peaks *I*_M1la_ (Fig. S3 A) and *I*_M1ha_ (Fig. S3 C) are higher at higher temperatures and in the presence of Dextran. The spacing of the M1_la_ reflection (*S*_M1la_; Fig. S3 B, filled circles) is 44.43 nm at 35°C in the presence of Dextran, and that of M1_ha_ (*S*_M1ha_; Fig. S3 D) is 42.16 nm (Table 1). The corresponding values for resting mouse EDL (triangles) are 44.74 and 41.76 nm. None of these spacings matches the fundamental axial periodicity of the myosin helix. Both *S*_M1la_ (Fig. S3 B) and *S*_M1ha_ (Fig. S3 D) decrease on cooling below 35°C; their temperature dependence is the opposite of that of the periodicity of the filament backbone reported by *S*_M6_ (Fig. 5 B).

The M2 reflection (Fig. 4 B) has four main sub-peaks in all conditions, plus additional side peaks or shoulders. These features are also present in resting intact muscle (Fig. 4 G). The four main peaks are not equally spaced; the lower-angle pair are more widely separated than the higher-angle pair, suggesting that the two pairs arise by x-ray interference from structures in each half-filament with distinct axial periodicities and filament locations. We therefore refer to the two lower-angle peaks as M2L and the two higher-angle peaks as M2H (Fig. 4 B). The centroid spacing *S*_M2H_ is 21.380 (±0.006) nm at 35°C in the presence of Dextran (Fig. S4 D and Table 1), corresponding to the second order of a fundamental H periodicity of 42.76 nm, close to the fundamental myosin-based repeat deduced above from *S*_ML1_ and *S*_M6_. *S*_M2L_ is 22.77 (±0.02) nm (Fig. S4 B), the second order of a fundamental L periodicity of 45.54 nm (Table 1). The L periodicity is clearly larger than the ∼43-nm periodicity associated with the myosin layer lines (*S*_ML1_) and the filament backbone periodicity (*S*_M6_) but similar to a previous estimate, 45.33 nm, from an analysis of the low-angle components of the M2, M3, M4, and M5 reflections from resting amphibian muscle (Oshima et al., 2012).

The intensities of M2L (*I*_M2L_; Fig. S4 A) and M2H (*I*_M2H_; Fig. S4 C) are higher at higher temperatures and in the presence of Dextran. These dependences are broadly similar to that of *I*_ML1_, except that the effect of Dextran on the M2L and M2H intensities remains large at 10°C. *S*_M2L_ and *S*_M2H_ are relatively independent of lattice compression by Dextran (Fig. S4, B and D) and decrease on cooling, like *S*_M1_. *S*_M2L_ is smaller in intact than in demembranated muscle at each temperature, but *S*_M2H_ is larger.

The intensities of the M4 and M5 reflections, like those of the M1 and M2, are larger at higher temperatures and in the presence of Dextran (Fig. 4). The M4 reflection has at least four sub-peaks, like the M2, but the M4L peaks are much weaker than the main M4H peak, which has a low-angle shoulder likely due to interference (Fig. 4 D). *I*_M4L_ is relatively insensitive to cooling (Fig. S5 A), but *I*_M4H_ (Fig. S5 C) has a strong temperature dependence similar to that of I_ML1_. The spacings of M4L (S_M4L_) and M4H (*S*_M4H_) are larger in demembranated fibers than in intact muscle, and the difference remains after lattice compression by Dextran (Figs. S5, B and D). Moreover, *S*_M4L_ increases on cooling in demembranated fibers but decreases on cooling in intact muscle (Fig. S5 B), and *S*_M4H_ decreases more markedly on cooling in intact muscle than in demembranated fibers (Fig. S5 D).

The L and H components of the M5 reflection are clearly separated (Fig. 4 F); the L sub-peaks are much weaker than the H sub-peaks and have a weaker temperature dependence (Fig. S6). *I*_M5H_ has dependencies on temperature and the presence of Dextran similar to that of *I*_ML1_. *S*_M5L_ and *S*_M5H_ are only weakly temperature dependent.

#### The M3 reflection

**Figure 6.**
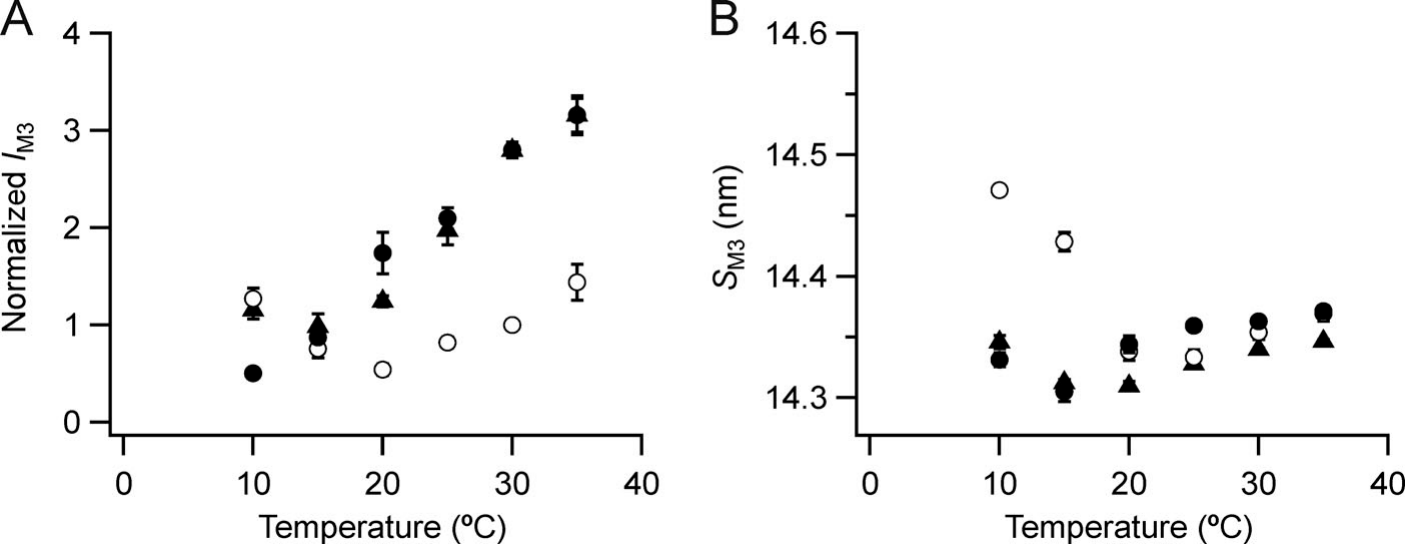
**Intensity and spacing of the M3 reflection.** **(A and B)** Intensity (A) and spacing (B) are shown. Mean ± SE. Open circles, no Dextran, *n* = 10 demembranated psoas fiber bundles; filled circles, 5% Dextran, *n* = 5; black triangles, resting intact mouse EDL muscles, *n* = 6. Intensities for demembranated fibers normalized by *I*_M3_ in the absence of Dextran at 30°C and those for intact muscles scaled to the value for demembranated fibers at 30°C in the presence of Dextran.

The M3 is not a forbidden reflection and stays strong during active contraction. Its spacing increases by ∼1.5% on activation, matching the spacing increase of the filament backbone signaled by the M6 (Huxley and Brown, 1967; Linari et al., 2000). Because the M3 reflection corresponds to the axial repeat of the myosin heads, it has been used extensively to obtain information about the conformation and movement of the heads in contracting muscle and during activation and relaxation (Piazzesi et al., 2007; Brunello et al., 2009; Reconditi et al., 2011). In relaxed demembranated fibers from rabbit psoas muscle at 35°C in the presence of Dextran, the M3 appears as a prominent lower-angle peak with a higher-angle shoulder (Fig. 4 C, red), similar to the profile in resting mouse EDL muscle at the same temperature (Fig. 4 G) and to that in resting amphibian muscle (Reconditi et al., 2011). Gaussian deconvolution of the M3 profile gives an estimate of the spacing of the most prominent peak as 14.410 ± 0.003 nm and that of the unresolved peak contributing to the high-angle shoulder as 14.206 ± 0.004 nm (Table S1). The centroid spacing of the M3 reflection (*S*_M3_) is 14.37 nm in these conditions (Fig. 6 B, filled circles; and Table 1), corresponding to the third order of a fundamental spacing of 43.11 nm, matching the myosin-based H periodicity signaled by the myosin layer lines (Fig. 3 D and Table 1). The reflection conventionally referred to as the M3 therefore corresponds to M3H according to the terminology used above. M3L peaks would be expected at reciprocal spacings of around 1/15.2 nm = 0.066 nm^−1^, and there is a characteristic set of three very weak peaks near that reciprocal spacing in both demembranated relaxed and intact resting muscle at 35°C (Fig. 4 G).

The effects of temperature and lattice compression on the main M3 reflection (M3H) in demembranated fibers are distinct from those on the other meridional reflections and myosin-based layer lines described above. The intensity of the M3 reflection (*I*_M3_; Fig. 6 A) is higher at higher temperatures and at 20°C and above is larger in the presence of Dextran (filled circles), but the shape of the temperature dependencies are quite different. *I*_M3_ has an almost linear dependence on temperature in the presence of Dextran but a biphasic relationship in its absence (open circles), with a minimum around 20°C, so that at 10°C *I*_M3_ is larger in the absence of Dextran.

The spacing of the M3 reflection (*S*_M3_; Fig. 6 B) also has a biphasic dependence on temperature, and the relationship in the presence of Dextran (filled circles) is similar to that in resting mouse EDL muscle (triangles), with a minimum of ∼14.30 nm at 15–20°C. There is no effect of lattice compression by Dextran on *S*_M3_ at 20–35°C but a large effect at 10°C, where *S*_M3_ in the absence of Dextran (open circles) is ∼0.8% larger than at 35°C. Thus, both in intact muscle and in demembranated muscle in the presence of Dextran, the effect of cooling on *S*_M3_, reporting the axial spacing of the myosin heads, is distinct from that on the filament backbone reported by *S*_M6_.

Cooling and lattice compression by Dextran have relatively little effect on the interference fine structure of the M3 reflection (Fig. 4 C), but at lower temperatures a new peak, corresponding to a spacing of ∼14.8 nm and designated by a star in Fig. 4 C, becomes prominent. The star peak is undetectable in demembranated fibers in the presence of Dextran at 35°C, but at low temperature is stronger in the presence of Dextran (Fig. S7 A). The spacing of the star peak (Fig. S7 B) decreases with increasing temperature, like that of M6 and with a similar functional dependence. The star peak is also present in resting intact mouse EDL muscle (triangles), with similar properties.

## Discussion

### Two axial periodicities in the thick filaments of skeletal muscle

The muscle thick filament was initially regarded as a rigid helical scaffold, with the simple function of presenting myosin motor or head domains at regular intervals on its surface for interaction with the surrounding actin filaments. However, early x-ray studies of resting amphibian muscle (Huxley and Brown, 1967) already established the existence of multiple meridional x-ray reflections corresponding to periodicities that did not match the ∼43-nm periodicity of the myosin-based helix. In particular, the low-angle component of the M1 reflection (M1_la_), corresponding to the longer axial periodicity of 44.4 nm (Table 1), became associated with MyBP-C following the report that it was intensified by labeling muscle fibers with an antibody to that protein (Rome et al., 1973).

**Figure 7.**
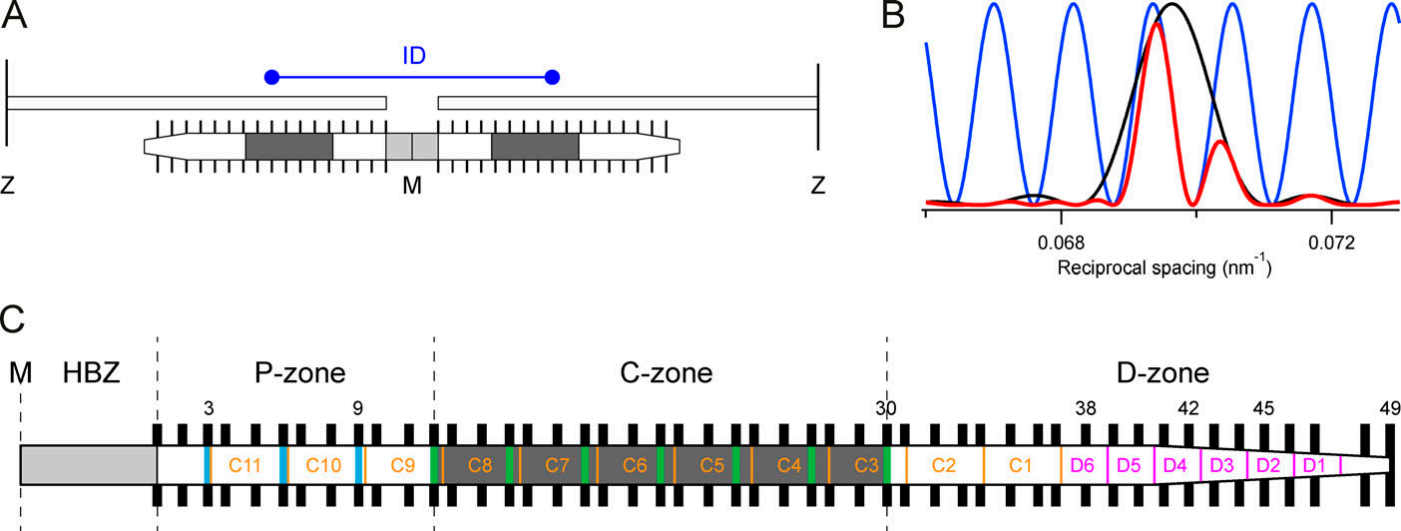
**Generation of x-ray interference sub-peaks by diffraction from the bipolar thick filament and schematic of the half-thick filament showing a possible structural basis for the H and L periodicities.** **(A)** The ordered layers of myosin motors (black bars) in each half-thick filament that contribute to the M3 x-ray reflection have mirror symmetry with respect to the M-line (M). Only one of every three layers of motors is shown for simplicity. The center-to-center distance between the ordered layers is the interference distance (ID, blue). The MyBP-C containing C-zones are shaded dark gray, and the bare zone which lacks myosin motors is light gray. Z, Z-lines defining the ends of the sarcomere and connecting the thin filaments. **(B)** Intensity distribution of the M3 reflection that would be produced by a single array of ordered motors in each half-filament (black), the fringes (blue) produced by interference between the two arrays in each filament, and the product of the black and blue functions, the resulting M3 profile (red), all calculated for relaxed demembranated fibers at 35°C in the presence of Dextran. **(C)** Detailed periodic structure of the half-filament. HBZ, half-bare zone. P-, C-, and D-zones, proximal, MyBP-C containing, and distal zones, respectively. Black bars, complete set of layers of myosin head domains numbered 1–49 from the M line. Green and blue vertical bars, MyBP-C and its paralogue proteins, respectively, assumed to align with “crown 1” of the myosin triplets in the C-zone (AL-Khayat et al., 2013). Orange, titin C-type super-repeats C1–C11 numbered in the direction of the primary sequence. The distal end of C11 was placed 110 nm from the M line (Tonino et al., 2019), the proximal end of C1 is then [110 + (11 * 45.54)] = 610 nm from the M line, assuming an L periodicity of 45.54 nm. Magenta, titin D-type super-repeats 1 to 6.

That association is complicated, however, by the fact that the M1_ha_ reflection, corresponding to a periodicity of ∼41.7 nm, was also enhanced by binding of the MyBP-C antibody and that all the meridional x-ray reflections are modulated by x-ray interference between the diffracting structures in each half-thick filament (Linari et al., 2000; Juanhuix et al., 2001; Huxley et al., 2006). X-ray interference effectively multiplies the diffraction pattern generated by the array in each half-filament by a fringe pattern with a periodicity related to the interference distance between the centers of the two diffracting arrays (Fig. 7 A), generating diffraction sub-peaks with spacings that are distinct from the physical periodicity of the arrays (Fig. 7 B). The separation between the interference sub-peaks is close to the effective spatial resolution achieved with the preparations, x-ray beam size, and detectors used in these experiments, so the interference sub-peaks are less clearly resolved in the corresponding experimental intensity profile (Fig. 4 C, red), although the reciprocal spacing and relative intensities of the sub-peaks can be determined by Gaussian deconvolution. However, the interpretation of the interference fine structure of lower-order reflections in particular is limited by spatial resolution.

Analysis of the interference sub-peaks of the M2 reflection illustrates the structural interpretation that is feasible at the current spatial resolution. The M2 is usually considered the second order of the 43-nm periodicity of the myosin helix, but it can now be resolved into four main sub-peaks plus additional side-peaks or shoulders, most clearly seen at near-physiological temperature in the presence of Dextran (Fig. 4 B, red). These sub-peaks are not equally spaced and therefore cannot be the result of multiplying a broad reflection from a single diffracting structure in each half-filament by a sinusoidal interference fringe pattern (Fig. 7 B). The simplest alternative interpretation, first suggested by Oshima et al. (2007, 2012) in their x-ray studies of resting amphibian muscle, is that the four main peaks are two pairs of interference sub-peaks from two structures with slightly different axial periodicities. We refer to these two pairs of sub-peaks of the M2 reflection as high-angle (M2H) and low-angle (M2L), respectively (Fig. 4 B), corresponding to the second-order reflections generated by two fundamental periodicities. The most accurate available estimate of the fundamental H periodicity is likely to be that from the spacing of the ML3 layer line (43.14 nm) because, unlike the meridionals, the layer lines are not influenced by interference. The best available estimate of the L periodicity (45.54 nm; Table 1) is that from the M2L sub-peaks, but this value should be regarded as approximate because of possible overlapping of the L and H sub-peaks or x-ray interference between the L and H structures, and a more precise estimate will require higher-resolution diffraction data. This limitation is illustrated in extreme form by the M1 reflection, the lower- and higher-angle components of which do not index on the L and H periodicities (Table 1), probably because each of these components contains unresolved interference sub-peaks.

Nevertheless, the values for the fundamental L and H periodicities given above are supported by analysis of higher-order meridional reflections (Table 1). Within the precision of the measurements, the M3, M4, M5, and M6 reflections all have strong components matching the expected orders of the fundamental H periodicity (solid vertical lines in Fig. 4, A–F). The M4 and M5 reflections have weaker L components (Fig. 4, A–F, dashed vertical lines); *S*_M5L_ matches the expected fifth order of the fundamental L periodicity, but *S*_M4L_ is lower than expected. The L components of the M3 and M6 reflections are very weak. The generally lower intensity of the higher-order L components suggests a broader mass distribution or greater disorder in the diffracting structure responsible for the L periodicity.

The H periodicity is likely to be generated by staggered axial packing between the coiled-coil tails of adjacent myosin molecules in the backbone of the filament (McLachlan, 1984). This conclusion is supported by the interference fine structure of the M3H reflection. The separation between the two most prominent sub-peaks of this reflection (Table S1) is 1/(1,003 ± 25 nm), matching that expected from distance between the centers of the 49 layers of myosin heads in each half-filament, 1/1,015 nm (see Materials and methods; Fig. 7 A; Linari et al., 2000). These distances are larger than the physical distance between the centers of the interfering arrays, which is 851 nm in this case, because of the systematic effect of modulation of the interference fringe pattern by the diffraction pattern produced by each individual array to decrease the sub-peak separation in reciprocal space (Fig. 7 B). The separation of the two sub-peaks of the M6H reflection is slightly larger, 1/(937 ± 48 nm), corresponding to a slightly shorter array of diffracting structures with the ∼7.2-nm periodicity in the filament backbone, in the region containing the first 45 layers of myosin heads. A shorter array of backbone diffractors is expected from the tapering of the filament at its tips, with fewer overlapping myosin tails in that region. The separation between the two M2H peaks (Table S1) is 1/(857 ± 10 nm), corresponding to roughly the first 42 layers of myosin heads. In contrast to the M3 and M6 reflections, the M2 is a forbidden reflection—it would be absent in the diffraction pattern from a perfect three-start helix and must be produced by regular perturbations of the helix, as observed in EM reconstructions of the cardiac thick filament (AL-Khayat et al., 2013), or additional nonhelical diffracting structures. These perturbations or extra structures must be confined to the inner region of each half-filament, and a similar estimate of their location, from layers 1 to 39, was made previously by modeling the meridional reflections from resting amphibian muscle (Malinchik and Lednev, 1992).

The L periodicity (ca 45.5 nm) is clearly longer than that of the myosin-based helix, and there is no detectable layer line with the L periodicity, so it must be associated with a nonhelical periodic structure. The interference sub-peaks of the M2L reflection are separated by 1/(637 ± 9 nm) at 35°C in the presence of Dextran (Table S1), close to the separation (1/634 nm) that would be produced by interference between nine repeats of the 45.5-nm L periodicity in each half-filament starting from the half-bare zone. The L reflections were previously identified with MyBP-C, initially prompted by the x-ray antibody-labeling studies mentioned above (Rome et al., 1973). Subsequently, two closely spaced axial periodicities were also identified in cryo-EM sections of skeletal muscle (Squire et al., 1982), and the longer periodicity was again suggested to be associated with MyBP-C. On the basis of these and other studies, the L reflections have sometimes been referred to as “C reflections” (Oshima et al., 2007, 2012).

However, there appear to be several difficulties with that assignment. The C-terminal domains of MyBP-C bind strongly to the myosin tail (Moos et al., 1975), and MyBP-C is too short to generate a distinct axial periodicity through its own end-to-end packing interactions. It has been suggested that the N-terminal domains of MyBP-C might take up a longer axial periodicity by binding to actin (Moos et al., 1978; Squire et al., 2003). However, the similar intensities of the M2L and M2H reflections (Fig. 4 B) seem difficult to reconcile with this hypothesis, given that there are 14 times more myosin heads than MyBP-C molecules in the filament and each head has a greater mass than the N-terminal MyBP-C domains. Moreover, the relative intensities of M2L and M2H are almost independent of interfilament spacing (Fig. 4 B), which might be expected to modulate binding of MyBP-C to actin as discussed below. Finally, stretching resting frog muscle fibers to decrease the overlap between the thick and thin filaments increases the intensity of M2L compared with that of M2H (Reconditi et al., 2014), the opposite of the change that would be expected if the L periodicity were generated by binding of MyBP-C to thin filaments.

The most likely alternative origin of the L periodicity is titin, the thick filament scaffold protein. The thick filament–binding region of titin is largely composed of repeating immunoglobulin/fibronectin domains, each ∼4 nm long, with distinct domain super-repeats in different regions of the thick filament (Fig. 7 C; Labeit and Kolmerer, 1995). The 11 “C-type” titin super-repeats, each containing 11 domains, extend from about layer 3 to 37 of the myosin heads, and the 6 distal or “D-type” super-repeats, each containing 7 titin domains, extend from about layer 37 to close to the filament tip (Tonino et al., 2019; Bennett et al., 2020). It has generally been assumed that each C-type titin super-repeat matches the ∼43-nm repeat of myosin, the H periodicity, and that the 11 C-type titin super-repeats overlap with 33 layers of myosin heads. However, if the 11 titin super-repeats overlapped with 35 layers of heads as diagrammed in Fig. 7 C (orange), their axial periodicity would be approximately (35/33) * 43.1 = 45.7 nm, close to the observed L periodicity. Note that in this hypothesis MyBP-C (Fig. 7 C, green stripes) follows the fundamental 43.1-nm H periodicity of myosin rather than the titin-based L periodicity. The systematic perturbations in the myosin crowns may also be extended to layers 3–37, the layers overlapping the C-type super-repeats (Fig. 7 C), rather than the conventional C-zone.

The origin of the L periodicity might in principle be identified by EM or super-resolution light microscopy of muscle samples labeled with antibodies to the candidate proteins. EM of the thick filament region of freeze-substituted fast skeletal muscle from amphibians shows 10 axial stripes with a periodicity of roughly 43 nm, the outer seven of which are also labeled with antibodies to MyBP-C (Luther et al., 2008, 2011). However, the spacing of these stripes cannot be measured precisely from the EM images because of unknown shrinkage during specimen preparation and sectioning. The axial periodicity of the thick filament region of titin was recently estimated by super-resolution fluorescence microscopy of isolated skeletal muscle myofibrils that had been glutaraldehyde fixed in rigor and labeled with antibodies to specific titin domains (Bennett et al., 2020). That study concluded that, averaged along the thick filament, the titin domain repeat was 3.98 nm, with 95% confidence limits of 3.92–4.03 nm, corresponding to an 11-domain super-repeat of 43.1–44.3 nm, slightly smaller than the 45.5-nm L periodicity measured here for demembranated or intact muscle fibers at 35°C in the presence of Dextran. It is not clear whether this difference can be accounted for by the different conditions or preparations used for the measurements.

The hypothesis that the L periodicity corresponds to the titin C-type super-repeat does not necessarily imply that the L reflections are generated by x-ray diffraction from the titin domains themselves. Given the strength of the L reflections, it is much more likely that the periodic structure responsible for the L reflections is a population of myosin head domains that adopt the L periodicity in the resting or relaxed state through their interaction with titin. For structural and stoichiometric reasons, the L-periodicity–binding sites would not be available to all the myosin heads, and the majority would retain the H periodicity generated by their tail packing and the surface helix. However, within each 43.1-nm H repeat, some individual heads or crowns are displaced from the positions they would occupy in a perfect 43.1-nm helix, giving rise to the forbidden meridional reflections as discussed above, and it is possible that some are further displaced to take up the L axial periodicity by binding to titin. Those L-periodicity myosin heads must have lost their helical order, since there is no detectable L layer line. For simplicity, we have not attempted to represent the hypothetical L-periodicity heads in Fig. 7 C, but within each 43.1-nm repeat, some individual heads would be displaced from the positions shown to align with the underlying C-type titin repeat (Fig. 7 C, orange).

### Effects of temperature on thick filament structure

The H and L periodicities of the thick filament are not constant but depend on experimental conditions, including temperature and interfilament spacing. These changes are not related to the elasticity of the thick filament (Reconditi et al., 2019); they occur in the absence of filament stress but mimic the structural changes associated with thick filament activation during contraction (Reconditi et al., 2011; Linari et al., 2015; Irving, 2017; Piazzesi et al., 2018; Caremani et al., 2019).

Cooling relaxed demembranated muscle fibers from 35°C to 10°C in the presence of 5% Dextran does not alter the lateral separation between the filaments (Fig. 2 C), so the effects of temperature in these conditions are independent of any indirect effects of changes in interfilament spacing. Cooling increases the equatorial intensity ratio *I*_11_/*I*_10_ (Fig. 2 D), suggesting that myosin heads move away from the thick filament backbone toward the thin filaments, although increased order of the thin filaments in the hexagonal lattice may also contribute to the observed change (Bershitsky et al., 2010). The intensity of the myosin layer lines is greatly reduced by cooling (Fig. 1 and Fig. 3), as reported previously for demembranated fibers in the absence of Dextran (Wray, 1987; Lowy et al., 1991; Malinchik et al., 1997; Xu et al., 1997; Wakabayashi et al., 1998; Bershitsky et al., 2010) and intact mammalian muscle (Caremani et al., 2019), suggesting that the movement of the motors away from the thick filament surface is linked to destabilization of the helical motor array on the filament surface. The change in the radial distribution of the first myosin layer line on cooling (Fig. 3 B) is consistent with motors moving away from the filament backbone.

To a first approximation, the intensity of the first myosin layer line (*I*_ML1_; Fig. 3 B) is expected to be proportional to the square of the relative number of myosin motors in the helical array. The square root of *I*_ML1_ (*A*_ML1_; Fig. S8, black circles) therefore gives an estimate of that number and suggests that cooling from 35°C to 10°C in the presence of Dextran reduces the number of helical motors by slightly more than 50%. A similar fractional reduction was estimated from the decrease in the intensity of the first myosin layer line on cooling of resting EDL muscles of the mouse (Caremani et al., 2019). The temperature dependence of the change in helical order of the myosin heads in relaxed demembranated fibers from rabbit psoas muscle estimated from *A*_ML1_ (Fig. S8, black circles) is also in good agreement with that of the orientation of probes on the RLC in the same preparation and solution conditions (Fig. S8, green circles; Fusi et al., 2015), in both the presence and absence of 5% Dextran.

The temperature dependence of the intensities of the forbidden meridional reflections (Fig. 4, Fig. S3, Fig. S4, Fig. S5, and Fig. S6) is broadly similar to that of *I*_ML1_. The intensities of the sub-peaks associated with the H and L periodicities decrease to a similar extent on cooling. The structures responsible for the forbidden reflections, axial perturbations of the helical myosin head array or axial periodic structures with the L and H periodicities, are lost in parallel with the weakening of the myosin helix. The spacing of the M6 reflection (*S*_M6_; Fig. 5 B) increases on cooling, indicating that the thick filament becomes slightly longer. All these cooling-induced structural changes in the thick filament are also associated with calcium activation at high temperature (Linari et al., 2015; Irving, 2017), but with larger amplitude. *S*_M6_, for example, increases by 1.4% on activation of mouse EDL muscle at 35°C (Caremani et al., 2019) but by only about half that amount on cooling of demembranated fibers to 10°C in the presence of Dextran. Cooling of resting or relaxed muscle at zero force induces structural changes in the thick filaments that are characteristic of maximum calcium activation, but the cooling-induced changes are of smaller amplitude.

Unexpectedly, the axial spacings of the lower-order meridional reflections do not follow the increase in those of the filament backbone on cooling reported by *S*_M6_ but actually decrease, by more than 2% in the case of *S*_M1ha_ (Fig. S3 D). The spacing of the M3 reflection from the myosin heads (*S*_M3_) has a complex intermediate behavior (Fig. 6 B), decreasing on cooling from 35°C to 20°C, both in demembranated fibers in the presence of Dextran and in intact EDL muscle, before increasing again on cooling below 20°C. The intensity of the M3 reflection has an almost linear dependence on temperature in the presence of Dextran (*I*_M3_; Fig. 6 A), in contrast with the sigmoidal dependence of x-ray signals like *I*_ML1_ and *S*_M6_ associated with activation of the thick filament. Below 25°C, a new peak (designated by a star in Fig. 4 C) appears on the low-angle side of the M3, with a spacing of ∼14.8 nm (Fig. S7 B), intermediate between the third-order spacings of the L and H periodicities described above. The star peak is also present in resting mouse EDL muscle at low temperature (Fig. S7 A; Caremani et al., 2019) and was previously observed in demembranated fibers from rabbit psoas muscle at 5°C in the absence of Dextran (Xu et al., 2006). Its origin is unknown, although its intermediate spacing suggests that it may be due to x-ray interference between the structures responsible for the L and H periodicities.

The distinct temperature dependences of the various x-ray signals described above show that changes in thick filament structure induced by cooling relaxed or resting muscle and by implication those associated with physiological activation cannot be simply explained by an extension of the filament backbone linked to weakening of the surface helix of myosin heads. Understanding of the additional structural changes induced by thick filament activation and their functional significance will depend on a more complete description of thick filament structure, including the multiple axial periodicities and their associated x-ray interference effects described in the previous section.

Cooling demembranated fibers from rabbit psoas muscle increases the sensitivity of the thin filaments to calcium (Stephenson and Williams, 1981). This effect is not accompanied by a change in the structure of the thin filaments in relaxing conditions as reported by the intensity or spacing of the T1 meridional reflection associated with ∼38-nm axial repeat of troponin (Fig. S9). Although there is no activation of the thin filament in the conditions of the present x-ray measurements at very low calcium concentration (pCa 9), the increased calcium sensitivity on cooling is consistent with the general hypothesis that the thick filament is more OFF in the helically ordered state at higher temperature, so a higher degree of thin filament activation is required in order to achieve the half-maximal force.

All these effects of cooling are likely to be linked to a change in the conformation of the myosin head domain associated with the ATP or switch-2 open structure of the head that is increasingly populated at lower temperatures (Wray, 1987; Malinchik et al., 1997; Xu et al., 1997, 1999, 2003). This head conformation appears to be less able to form the inter- or intra-molecular interactions that stabilize the helically ordered OFF state and the associated shorter filament backbone periodicity. As we discussed elsewhere (Caremani et al., 2019), comparison of the structural and functional effects of cooling on resting and isometrically contracting intact EDL muscles suggests that the helically ordered OFF or switch-2 closed state is required for entry into the active force-generating cross-bridge cycle in the presence of calcium.

### Effects of interfilament spacing on thick filament structure

Osmotic compression of the filament lattice of demembranated fibers from rabbit psoas muscle by 5% Dextran results in an interfilament spacing slightly smaller than that in intact EDL muscles of the mouse (Fig. 2 C). Many published studies of demembranated rabbit psoas muscle fibers have been performed at 10°C or less in the absence of Dextran, conditions in which the lattice spacing parameter *d*_10_ would be at least 43 nm, much larger than the value, ∼34 nm, in near-physiological conditions in intact muscle or in demembranated muscle in the presence of 5% Dextran (Fig. 2 C). Taking the radii of the thick and thin filaments as ∼9 and 5 nm, respectively, the closest surface-to-surface distance between the filaments in the 1,1 planes at 10°C in the absence of Dextran is ∼15 nm, almost twice that in near-physiological resting or relaxed conditions, ∼8 nm.

In addition to possible direct effects on interactions between myosin heads and thin filaments, a larger interfilament spacing might reduce the extent of binding of the N-terminal domains of MyBP-C to thin filaments. The fast skeletal muscle isoform of MyBP-C is composed of 10 immunoglobulin or fibronectin type-3 domains, each ∼4 nm in diameter. N-terminal domains 1 and 2 can bind to the thin filament, and C-terminal domains 8 to 10 are likely to be bound to the thick filament, leaving at most five domains, with a maximum end-to-end distance of ∼20 nm, to bridge the inter-filament gap. The increase in the size of the gap from 8 to 15 nm estimated above might therefore reduce the fraction of MyBP-C N-terminal domains bound to thin filaments.

The intensities of all the myosin-based reflections, including that of the first myosin layer line (*I*_ML1_; Fig. 3 C) and of the M1–M6 meridionals including the forbidden reflections and their sub-peaks (Fig. 4), are weaker in the absence of Dextran at 35°C. The magnitude of the effect is similar for all these reflections, including the M6 associated with the axial periodicity of the filament backbone. The troponin-based T1 reflection is also somewhat weaker in the absence of Dextran (Fig. S9 B). Lattice compression seems to have a general effect of enhancing the meridional intensities at 35°C, making it difficult to isolate any specific effect on the helical or axial order of the myosin head domains. This effect could be at least partly explained by the increased number of filaments illuminated by the x-ray beam after compression of the filament lattice. There is no clear effect of lattice compression on the radial peak of the first myosin layer line at 35°C (Fig. 3 B). However, the 1,0 equatorial reflection was weaker in the presence of Dextran at all temperatures studied (Fig. S1 A), and the 1,1 reflection was weaker at low temperatures (Fig. S1 B). Those effects were reported previously (Malinchik and Yu, 1995) and can be explained as a consequence of the change in lattice spacing, with a fixed shell of myosin heads on the surface of the thick filaments at the higher temperatures, consistent with the absence of a change in the radial distribution of ML1 on lattice compression in these conditions. The axial periodicity of the filament backbone (*S*_M6_) is ∼0.3% larger in the absence of Dextran at 35°C (Fig. 5 B), signaling a slightly more ON structure, but there is no clear effect on the spacing of the myosin layer lines (Fig. 3 D) or on that of the M3 reflection (Fig. 6 B). In summary, the present results provide no compelling evidence for an effect of lattice compression at near-physiological temperature on the conformation of the myosin motors, but there is a small effect on the filament backbone that may be mediated by an unresolved change in the conformation of myosin motors or MyBP-C.

The effects of lattice compression by 5% Dextran are much more prominent at lower temperatures, when there is also a larger change in *d*_10_ (Fig. 2 C). These effects are most clearly seen in *I*_ML1_ (Fig. 3 C), *S*_ML1_ (Fig. 3 D), *S*_M6_ (Fig. 5 B), and *S*_M3_ (Fig. 6 B). In each case, the transition to a more ON structure induced by cooling is inhibited by lattice compression; the transition temperature is much lower in the presence of lattice compression. In general, these effects show that the OFF structure of the thick filament is stabilized at lower interfilament spacing. A similar conclusion was deduced from studies of the orientation of probes on the RLC of myosin (Fig. S8; Fusi et al., 2015).

Compression of the filament lattice by Dextran sensitizes the thin filaments to calcium at lower temperature (Godt and Maughan, 1981); in the conditions of the present experiments, the [Ca^2+^] required for half-maximal isometric force is reduced by ∼0.1 pCa units at 12°C (Fig. S10). Thus, although increasing the temperature and compressing the filament lattice both stabilize the OFF state of the myosin filament at pCa 9, they have opposite effects on the calcium sensitivity of the thin filament. This apparent paradox might be resolved by the hypothesis, deduced from the correlation in intact mouse EDL muscle between the fraction of myosin motors in the OFF state at different temperatures with the fraction that bind to actin on calcium activation, that only the OFF motors can be recruited by calcium activation, the others being in a refractory state at low temperature (Caremani et al., 2019). However, that mechanism may not operate in demembranated fibers from rabbit psoas muscle, as discussed below, and an alternative explanation for the effect of lattice compression on calcium sensitivity would be that binding of the N-terminal domains of MyBP-C to the thin filaments increases the calcium sensitivity of force and thin filament activation in both skeletal and cardiac muscle (Kunst et al., 2000; Kampourakis et al., 2014). Increasing the number of MyBP-C cross-links by lattice compression seems to have the dual effect of calcium-sensitizing the thin filament at activating [Ca^2+^] while stabilizing the OFF structure of the thick filament at low [Ca^2+^], a combination that could in principle promote a doubly inhibited OFF state in resting muscle to minimize ATP utilization, combined with a rapid cooperative response of both filaments to an activating pulse of calcium.

### Effects of demembranation of muscle cells on thick filament structure

Demembranation of muscle fibers has been used in many previous studies to control the constituents of the solution bathing the contractile filaments, in particular free [Ca^2+^] and the concentrations of ATP and its hydrolysis products, or the protein composition of the filaments themselves. Many features of the x-ray diffraction patterns from demembranated fibers of rabbit psoas muscle at 35°C in the presence of 5% Dextran match those of intact resting EDL muscles of the mouse at the same temperature, as most clearly shown by comparison of the meridional intensity distributions (Fig. 4 G). The fundamental L and H periodicities are the same in the two preparations within the precision of the calibrations (Table 1), although the L reflections are slightly more intense in intact muscle and there are some systematic differences in the spacings of the L and H components of the M2 and M4 reflections. The interference fine structure of the meridional reflections, including the relative intensities and separations of the respective sub-peaks, is also similar, suggesting that the conformations and filament locations of the diffracting structures are also similar.

The effect of cooling to increase the axial spacing of the filament backbone is larger in demembranated fibers (*S*_M6_; Fig. 5 B), and this difference is shown more clearly by plotting the relative amplitude of the first myosin layer line (*A*_ML1_), a signal of the regulatory state of the myosin heads, against *S*_M6_, a signal for the regulatory state of the filament backbone, for the different temperatures (Fig. S11). The slope of this relationship is lower in demembranated (circles) than intact muscle (triangles) and lower in the absence of Dextran (open circles), suggesting that the coupling between the backbone and head conformations has been weakened by demembranation.

The relative intensity of the forbidden and nonforbidden reflections (e.g., the ratio of *I*_M2_ to *I*_M3_) is much greater in demembranated than intact fibers, even at 35°C (Fig. 4 G), suggesting that the axial perturbations of the myosin helix are considerably larger after demembranation. The ratio of the intensities of the 1,1 and 1,0 equatorial reflections (*I*_11_/*I*_10_; Fig. 2 D) is slightly lower in demembranated than intact muscle; this does not seem to be due to a lower mean radius of the myosin heads in the demembranated fibers, since the radial profile of the first myosin layer line indicates a slightly higher radius in that preparation (22 versus 20 nm). The lower value of *I*_11_/*I*_10_ in demembranated fibers might be associated with a slightly greater degree of disorder of the thin filaments in the hexagonal lattice (Malinchik and Yu, 1995; Bershitsky et al., 2010).

Apart from the larger axial perturbations of the myosin helix and increased lateral disorder of the thin filaments, other aspects of the high-temperature OFF structure of filament structure in resting intact muscle are largely preserved in relaxing conditions after demembranation, provided that the filament lattice is compressed by 5% Dextran. This may be considered a surprising result, given the loss of many soluble protein components of the muscle cytoplasm and reduction of the molecular crowding characteristic of the intact muscle cell, replacement of the solution bathing the filaments by an artificial relaxing solution, and largely uncharacterized changes in posttranslational modifications of filament proteins including the myosin RLC, MyBP-C, and troponin. The comparison is limited by the different muscle types and species of the demembranated and intact muscle fiber preparations. The effects of these individual factors have not yet been characterized. However, many aspects of thick filament structure are preserved after demembranation in the presence of 5% Dextran, even at 25°C, a temperature that is more experimentally tractable for studies of actively contracting mammalian muscle.

An important limitation of the present structural results is that they were all obtained at low [Ca^2+^] and cannot necessarily be extrapolated to functional studies at activating [Ca^2+^]. For example, the effect of cooling to reduce the number of actin-bound force-generating myosin heads in intact EDL muscle (Caremani et al., 2019) is not present in demembranated fibers from rabbit psoas muscle (Bershitsky and Tsaturyan, 2002; Linari et al., 2007). Further studies will be required to determine the effects of demembranation on thick filament structure at activating [Ca^2+^]. That caveat notwithstanding, the great majority of published studies on demembranated muscle fibers have been made at low temperatures in the absence of lattice compression, conditions in which the native structure of myosin and the thick filaments in relaxing conditions, and by implication the myosin-based regulatory mechanisms, are severely disrupted.

### Implications for understanding the regulation of muscle contraction

The present results extend previous x-ray studies of thick filament regulation in intact muscle fibers (Linari et al., 2015; Caremani et al., 2019) to a widely used demembranated muscle preparation in which the interfilament spacing can also be controlled. They are complementary to previous studies of thick filament structure using bifunctional fluorescent probes in the same demembranated muscle preparation (Fusi et al., 2015, 2016), but add the extensive structural information encoded in the low-angle x-ray diffraction pattern (Fig. 1). The x-ray reflections cannot yet be interpreted in terms of an atomic model of the thick filament, but the analysis presented here takes some steps in that direction in a proposed annotation of two distinct axial periodicities in the thick filament. In near physiological conditions, also obtained in the demembranated fibers in the presence of 5% Dextran, the H periodicity is the well-known 43.1-nm helical periodicity of the myosin head domains, also responsible for the much-studied M3 reflection from the axial repeat of the myosin head domains and the M6 reflection that is dominated by periodicities in the thick filament backbone (Linari et al., 2000; Huxley et al., 2006; Piazzesi et al., 2007; Reconditi et al., 2011; Linari et al., 2015). The L periodicity, ∼45.5 nm, is purely axial, and we have suggested a possible structural basis for that periodicity in the C-type super-repeats of titin.

As understanding of the structure of the thick filaments from mammalian skeletal muscle has advanced, it has become increasingly clear that the original concept of the thick filament as a simple scaffold to hold the myosin head domains in suitable locations for interacting with the thin filaments and transmit the active force generated by those motors should be amended to include an additional role as a regulatory element, a role that was originally considered to be confined to the thin filament. Moreover, the original idea that all 294 motors in each half-filament are functionally equivalent also appears to be an oversimplification; different regions of the half-filament have different structures and may have different functions. Activation of the thick filament during physiological contraction may not be complete in either skeletal muscle or heart muscle (Piazzesi et al., 2018). The extent of activation of the thick filaments in heart muscle depends on the load (Reconditi et al., 2017), and different regions of the half-thick filament are activated during different phases of the contraction (Brunello et al., 2020). X-ray interference provides a uniquely powerful tool to investigate these regional differences in intact muscle sarcomeres.

The experiments reported here used variations of temperature and interfilament spacing to control the regulatory state of the thick filaments in relaxed or resting muscle in the absence of filament stress or activating calcium. Despite that limited scope, one conclusion of broad significance for the design of future studies and the interpretation of the many published studies of muscle regulation in situ is that many features of the physiological OFF structure of the thick filament are largely preserved after demembranation in relaxing conditions, but only at temperatures of 25°C and above. Other features, notably the characteristic perturbation of the myosin helix, are altered by demembranation. Determining whether these conclusions can be extended to activating concentrations of calcium has demanding implications because prolonged activation of demembranated muscle fibers in that temperature range leads to sarcomere disorder and irreversible damage, necessitating the use of temperature-jump or photolytic activation protocols (Goldman et al., 1987; Linari et al., 2007; Fusi et al., 2014).

Even the most extreme conditions used in the present study, cooling to 10°C in the absence of Dextran, produced only partial activation of the thick filaments in relaxing conditions at pCa 9, in comparison with full activation at physiological temperature. Nevertheless, the results show that thick filament activation cannot be simply described as a loss of the helical order of the myosin heads tightly coupled to extension of the filament backbone, and they start to provide some insight into the role of the different filament regions and their accessory proteins, especially MyBP-C, in the regulatory transition. They also provide further annotation of the x-ray diffraction pattern to identify x-ray signals for the different structural aspects of filament activation that can be exploited in future studies.

Cooling mammalian muscle is likely to produce its activating effect by increasing the number of myosin heads in the ATP or switch-2 open state as described previously, presumably because that motor conformation is less favorable for the helical packing of the heads associated with their OFF state. Lattice compression, which, in the conditions studied here, can decrease the surface-to-surface distance between the thick and thin filaments from ∼15 to 8 nm, stabilizes the OFF state of the thick filament against activation by the increased proportion of ATP heads in relaxing conditions at low temperature. This stabilization may be mediated by an increased probability that the N-terminal domains of MyBP-C bind to thin filaments in the compressed lattice, although so far there is no direct evidence for such a change. Moreover, the increased calcium sensitivity of active force generation following lattice compression suggests that such increased binding of the N-terminal domains of MyBP-C to thin filaments may also have an activating effect at higher [Ca^2+^].

A general limitation of the present experiments is that they were confined to relaxed or resting muscle at very low calcium concentrations. It will be important in future work to extend these studies to higher [Ca^2+^], including full calcium activation in order to understand how the changes in thick filament structure described here at different temperatures and interfilament spacings relate to those induced by calcium activation. Those experiments will need to employ protocols for rapid activation of mammalian muscle fibers at high temperature. Continuing enhancement of synchrotron x-ray sources together with advanced pixel array detectors will make such experiments both feasible and more informative.

## Supplementary Material

Table S1 reports the spacing of the component peaks of the M2, M3, and M6 meridional reflections at 35°C in demembranated bundles of psoas muscle fibers in the presence of 5% Dextran T500 and in intact EDL muscle at rest.
