# Supplementary material for: Dependence of thick filament structure in relaxed mammalian skeletal muscle on temperature and interfilament spacing
Source: J Gen Physiol. 2021 Jan 8;153(3):e202012713. doi: 10.1085/jgp.202012713 (PMC7802359; doi:10.1085/jgp.202012713)
Supplement: Table S1 — reports the spacing of the component peaks of the M2, M3, and M6 meridional reflections at 35°C in demembranated bundles of psoas muscle fibers in the presence of 5% Dextran T500 and in intact EDL muscle at rest. [file JGP_202012713_TableS1.docx]

Table S1. Spacing of the component peaks of M2, M3, and M6 meridional reflections at 35°C in demembranated bundles of psoas muscle fibers in the presence of 5% Dextran T500 and in intact EDL muscle at rest

|  | | Demembranated relax |  | Intact resting |
| --- | --- | --- | --- | --- |
|  |  | Spacing ± SE (nm) [*n*] |  | Spacing ± SE (nm) [*n*] |
| M2L | la | 23.800 ± 0.014 [4] |  | 23.840 ± 0.017 [3] |
|  | ma | 23.121 ± 0.012 [4] |  | 23.173 ± 0.005 [3] |
|  | ha | 22.311 ± 0.005 [4] |  | 22.257 ± 0.007 [3] |
| M2H | la | 21.624 ± 0.004 [4] |  | 21.563 ± 0.003 [3] |
|  | ha | 21.092 ± 0.005 [4] |  | 21.062 ± 0.011 [3] |
| M3H | la | 14.608 ± 0.004 [4] |  | 14.539 ± 0.005 [6] |
|  | ma | 14.410 ± 0.003 [4] |  | 14.360 ± 0.002 [6] |
|  | ha | 14.206 ± 0.004 [4] |  | 14.160 ± 0.003 [6] |
| M6H | la | 7.205 ± 0.002 [3] |  | 7.1957 ± 0.0004 [3] |
|  | ha | 7.151 ± 0.002 [3] |  | 7.1398 ± 0.0005 [3] |

SE, standard error.
